# SLUG/SNAI2 and Tumor Necrosis Factor Generate Breast Cells With CD44+/CD24- Phenotype

**DOI:** 10.1186/1471-2407-10-411

**Published:** 2010-08-06

**Authors:** Poornima Bhat-Nakshatri, Hitesh Appaiah, Christopher Ballas, Patricia Pick-Franke, Robert Goulet, Sunil Badve, Edward F Srour, Harikrishna Nakshatri

**Affiliations:** 1 Department of Surgery, Indiana University School of Medicine, West Walnut Street, Indianapolis, IN 46202, USA; 2 Department of Medicine, Indiana University School of Medicine, West Walnut Street, Indianapolis, IN 46202, USA; 3 Department of Pathology, Indiana University School of Medicine, Barnhill Drive, Indianapolis, IN 46202, USA; 4 Department of Biochemistry and Molecular Biology, Barnhill Drive, Indiana University School of Medicine, Indianapolis, IN 46202, USA

## Abstract

**Background:**

Breast cancer cells with CD44+/CD24- cell surface marker expression profile are proposed as cancer stem cells (CSCs). Normal breast epithelial cells that are CD44+/CD24- express higher levels of stem/progenitor cell associated genes. We, amongst others, have shown that cancer cells that have undergone epithelial to mesenchymal transition (EMT) display the CD44+/CD24- phenotype. However, whether all genes that induce EMT confer the CD44+/CD24- phenotype is unknown. We hypothesized that only a subset of genes associated with EMT generates CD44+/CD24- cells.

**Methods:**

MCF-10A breast epithelial cells, a subpopulation of which spontaneously acquire the CD44+/CD24- phenotype, were used to identify genes that are differentially expressed in CD44+/CD24- and CD44-/CD24+ cells. Ingenuity pathway analysis was performed to identify signaling networks that linked differentially expressed genes. Two EMT-associated genes elevated in CD44+/CD24- cells, SLUG and Gli-2, were overexpressed in the CD44-/CD24+ subpopulation of MCF-10A cells and MCF-7 cells, which are CD44-/CD24+. Flow cytometry and mammosphere assays were used to assess cell surface markers and stem cell-like properties, respectively.

**Results:**

Two thousand thirty five genes were differentially expressed (p < 0.001, fold change ≥ 2) between the CD44+/CD24- and CD44-/CD24+ subpopulations of MCF-10A. Thirty-two EMT-associated genes including SLUG, Gli-2, ZEB-1, and ZEB-2 were expressed at higher levels in CD44+/CD24- cells. These EMT-associated genes participate in signaling networks comprising TGFβ, NF-κB, and human chorionic gonadotropin. Treatment with tumor necrosis factor (TNF), which induces NF-κB and represses E-cadherin, or overexpression of SLUG in CD44-/CD24+ MCF-10A cells, gave rise to a subpopulation of CD44+/CD24- cells. Overexpression of constitutively active p65 subunit of NF-κB in MCF-10A resulted in a dramatic shift to the CD44+/CD24+ phenotype. SLUG overexpression in MCF-7 cells generated CD44+/CD24+ cells with enhanced mammosphere forming ability. In contrast, Gli-2 failed to alter CD44 and CD24 expression.

**Conclusions:**

EMT-mediated generation of CD44+/CD24- or CD44+/CD24+ cells depends on the genes that induce or are associated with EMT. Our studies reveal a role for TNF in altering the phenotype of breast CSC. Additionally, the CD44+/CD24+ phenotype, in the context of SLUG overexpression, can be associated with breast CSC "stemness" behavior based on mammosphere forming ability.

## Background

Cancer stem cell theory proposes that cancers may arise from malignant transformation of normal stem/progenitor cells. Alternatively, cellular plasticity and/or the tumor microenvironment may permit mature/differentiated cells to acquire a stem/progenitor phenotype [1-6]. Tumorigenic stem/progenitor cells have been documented in hematologic malignancies as well as in solid tumors, although correct terminology for these cells (cancer stem cells versus tumor initiating cells) is still a matter of debate [7-9]. Several studies implicate a subset of human breast cancer cells with an enhanced ability to form tumors in immunocompromised mice [10,11]. This subpopulation of cells also demonstrated the capacity for self-renewal and generation of heterogeneous progeny. At present, two distinct cell types have been described as CSCs for breast cancer. Cancer cells that display the cell surface marker profile of CD44^+^/CD24^-^/Lineage^- ^were the first described tumorigenic progenitor cell types for breast cancer [10]. NOD/SCID mice implanted with as few as 200 CD44+/CD24- cells form tumors. In addition, disseminated cancer cells in bone marrow with CD44+/CD24- phenotype have been identified in patients, although the prognostic relevance of this is as yet unclear [12-14]. Signaling pathways implicated in self-renewal and survival of normal organ-specific stem cells and embryonic stem cells, such as Hedgehog, Notch and Wnt/β-catenin, may be involved in maintaining "stemness" of CD44+/CD24-/lineage- cells [15-18]. The gene expression pattern of CD44+/CD24- CSCs is more similar to normal CD44+/CD24- breast epithelial cells than to CD44-/CD24+ cells isolated from tumors [19]. Recent studies have demonstrated enrichment of CSC gene expression signature in breast cancers that are classified as Claudin-low subtype [20]. We demonstrated that breast cancer cells with CD44+/CD24- phenotype express elevated levels of invasion-associated genes and are invasive but this phenotype is not a requisite for homing and growth at sites of metastasis [21]. In subsequent studies, normal and cancerous breast epithelial cells expressing higher levels of aldehyde dehydrogenase 1 (ALDH1) were described as normal and tumorigenic stem/progenitor cells [22]. Functional assays revealed ALDEFLUOR-positive cells (aldefluor staining provides indirect estimation of all ALDHs in cells) to be highly tumorigenic relative to ALDEFLUOR-negative cells. Moreover, the most tumorigenic phenotype identified was ALDEFLUOR+/CD44+/CD24- cells [22]. Additional refinement of the breast cancer stem cell phenotype has been described recently [23].

During our analysis of breast cancer cell lines for subpopulations with the CD44+/CD24- phenotype, we observed that almost all cell lines with a CD44+/CD24- subpopulation were basal breast cancer cells that had undergone epithelial to mesenchymal transition (EMT) [21]. Others have also reported enrichment of cells with the CD44+/CD24- phenotype in basal-like breast tumors [14]. EMT is a developmental process during which epithelial cells acquire a fibroblastoid and invasive phenotype, down-regulate epithelial-specific proteins, and induce various mesenchymal proteins [24]. There are specific changes in the gene expression profile during EMT. These include expression of vimentin and loss of E-cadherin expression; the change in expression of both of these markers has been associated with poor prognosis in breast cancer [25-27]. Activation of oncogenic and receptor tyrosine kinase pathways such as Ras and Src, or signaling through transforming growth factor beta (TGFβ), hepatocyte growth factor (HGF) and platelet derived growth factor (PDGF) can trigger EMT [24,28]. These signaling pathways induce the expression of the SNAIL family of transcription repressors, which reduce E-cadherin expression [29]. SNAIL family members involved in initiation and/or maintenance of EMT include SNAIL-1 [30,31], Snai2/SLUG [32,33], E12/E47 [34], ZEB-1/ZFHX1A [35], ZEB-2/ZFXH1B/Smad-Interacting Protein (SIP1) and TWIST [36].

Recent studies have shown that some members of the SNAIL family confer an EMT phenotype to breast epithelial cells, which correlates with cells changing phenotype from CD44-/CD24+ to CD44+/CD24- [37,38]. However, it is not known whether all genes that induce EMT confer a CD44+/CD24- phenotype to breast epithelial cells or if all breast epithelial subtypes are equally susceptible to such EMT-mediated phenotypic change.

In this study we have utilized the basal cell phenotype MCF-10A breast epithelial cell line [39] to study the association between CD44+/CD24- and the EMT phenotype. Although the majority of MCF-10A cells are CD44-/CD24+ or CD44+/CD24+, a fraction of these cells are CD44+/CD24-. Gene expression analysis of CD44+/CD24- cells compared to CD44-/CD24+ cells revealed increased expression of 32 EMT associated genes including SLUG, ZEB-1, ZEB-2, Hedgehog signaling associated gene Gli-2, and the metastasis-associated gene SATB-1. Transgenic overexpression studies showed that only SLUG had the capacity to alter the phenotype of CD44-/CD24+ MCF-10A cells to induce a subpopulation of CD44+/CD24- cells. However, transgenic overexpression of SLUG in the luminal type breast cancer cell line, MCF-7, generated cells with a CD44+/CD24+ phenotype, suggesting that basal cell types but not luminal cell types are susceptible to EMT associated acquisition of CD44+/CD24- phenotype. Additionally, only specific EMT associated genes induced a CD44+/CD24- phenotype in MCF-10A cells. For example, overexpression of the NF-κB subunit of p65 upregulates expression of ZEB-1 and ZEB-2 genes [40], and this resulted an increase in the percent of CD44+/CD24+ MCF-10A cells but not the percent of CD44+/CD24- cells.

## Methods

### Cell lines, plasmids, reagents, and retroviral vector transduction

MCF-7, MCF-10A and Ampho-phoenix cells were maintained as described previously [40,41]. All experiments were done with cells of similar confluence, except in case of TNF treated cells, which were always less dense compared to control cells at the time of harvest. MCF-10Ap65 cells have been described previously [40]. The bicistronic retrovirus vector pcQXIP (Clontech, CA) was used to generate MCF-10A cells overexpressing Ras (Clontech, CA). SLUG and Gli-2 cDNAs were generous gifts from Drs. E. R. Fearon (University of Michigan) [33], and H. Sasaki (Osaka University) [42], respectively. SATB-1 cDNA was purchased from Origene Technologies, Inc (Rockville, MD). The SLUG and SATB-1 cDNA was cloned into pcQXIN to generate supernatant containing retroviral vector. Packaging of retroviral vector particles, transduction, and selection of transduced cells have been described previously [40,41]. Tumor necrosis factor (TNF) was purchased from R&D Systems (Minneapolis, MN, USA).

### Matrigel and Mammosphere assays

Matrigel assay was performed as described previously [40]. Briefly, cells were plated on matrigel in eight-well chamber slides for 10 days. Matrigel was fixed overnight in formalin and embedded on histogel as described previously [40]. For the mammosphere assay, one hundred thousand cells were plated on ultralow adherent plates in serum free media with methylcellulose and supplements as described previously [38]. After 10 days of culturing, mammospheres in 10 independent microscopic fields were enumerated. Alternatively, media with mammospheres were collected, washed in PBS, resuspended in two ml PBS and large colonies were counted using a hemocytometer and expressed as number of mammospheres per 100,000 cells.

### Electrophoretic mobility shift assay (EMSA)

Whole cell lysates prepared from MCF-10A, CD44+/CD24-, and CD44-/CD24+ cells were subjected to EMSA as described previously [43]. For supershift assays, DNA:protein complexes were incubated with antibodies for ten minutes before electrophoresis. An antibody against p65 was from Millipore Corporation (Billerica, MA, USA), whereas p50 and p52 antibodies were from Santa Cruz Biotechnology (Santa Cruz, CA, USA). DNA binding activities of Oct-1 and SP-1 in these extracts were measured as controls. All oligonucleotide probe DNAs were purchased from Promega Corporation (Madison, Wisconsin, USA).

### Flow cytometric sorting of CD44+/CD24- and CD44-/CD24+ MCF-10A cells and additional flow cytometry analyses

Cells were incubated with FITC conjugated CD44 and PE conjugated CD24 antibodies (BD Biosciences, San Jose, CA, Catalogue number 555478 and 558428, respectively) as described previously [21]. Cells were sorted by flow cytometry and sorted cells were grown in culture for three days before RNA preparation. The same antibodies were used for characterizing cells under different treatment conditions. Note that all flow cytometry data presented here included isotype controls, individual antibody, and combination of antibodies. To conserve space, data from CD44 and CD24 antibody combination only are presented in some figures.

### Microarray analysis

For each experimental condition, four independent samples of total RNA were prepared using RNAeasy kits (Qiagen, Valencia, CA, USA) according to the manufacturer's instructions. Microarray hybridization using Affymetric Human Genome HG 133 plus 2 GeneChip and statistical analysis was performed as previously described [44]. Briefly, data were extracted using the MicroArray Suite 5 (MAS5) algorithm and exported into MicroArray Data Portal for analysis and linking to bioinformatics resources. Prior to analysis, probe sets that were not called present in at least half of the arrays in at least one experimental condition were removed; this procedure reduced the number of false positives. The MAS5 signals (expression values) were log transformed, and differences in gene expression between CD44+/CD24- and CD44-/CD24+ cells were identified by Welch's *t*-tests. The gene expression array data have been submitted to the Gene Expression Omnibus (GEO) database http://www.ncbi.nlm.nih.gov/geo/ under the accession number GSE15192.

### Reverse transcription polymerase chain reaction (RT-PCR)

The one step RT-PCR kit from Invitrogen Corporation (Carlsbad, CA, USA) was used to perform RT-PCR according to the manufacturer's instructions. RT-PCR (Usually at 60°C annealing and 72°C amplification) cycles varied between primer sets to ensure that amplification has not reached saturation level. Quantitative RT-PCR using SyberGreen on a TaqMan 7900HT instrument (Applied Biosystems, Carlsbad, CA, USA) was performed according to the manufacturer's instructions in order to measure SLUG expression in MCF-10A cells. Additional file 1, Table S1 contains the primer sequences used.

### Antibodies and Western blot analysis

Antibodies against E-cadherin (BD Biosciences), N-cadherin (Cell Signaling Technologies, Danvers, MA, USA), vimentin (Sigma, St. Louis, MO, USA), alpha smooth muscle actin (αSMA) (Sigma), and beta-actin (Sigma) were used for western blot analyses as per instructions from manufacturers. Cell lysate preparations in radioimmunoassay buffer and blotting conditions have been described previously [40].

### Cell proliferation assay

Cell proliferation assays were performed in 96-well plates and rate of proliferation was measured using bromodeoxyuridine-ELISA assay (EMD Biosciences, San Diego, CA, USA). One thousand cells were plated on day one and proliferation rate was measured at days four and six.

## Results

### Significant gene expression differences between the CD44+/CD24- and CD44-/CD24+ subpopulations of MCF-10A cells

**Figure 1:**
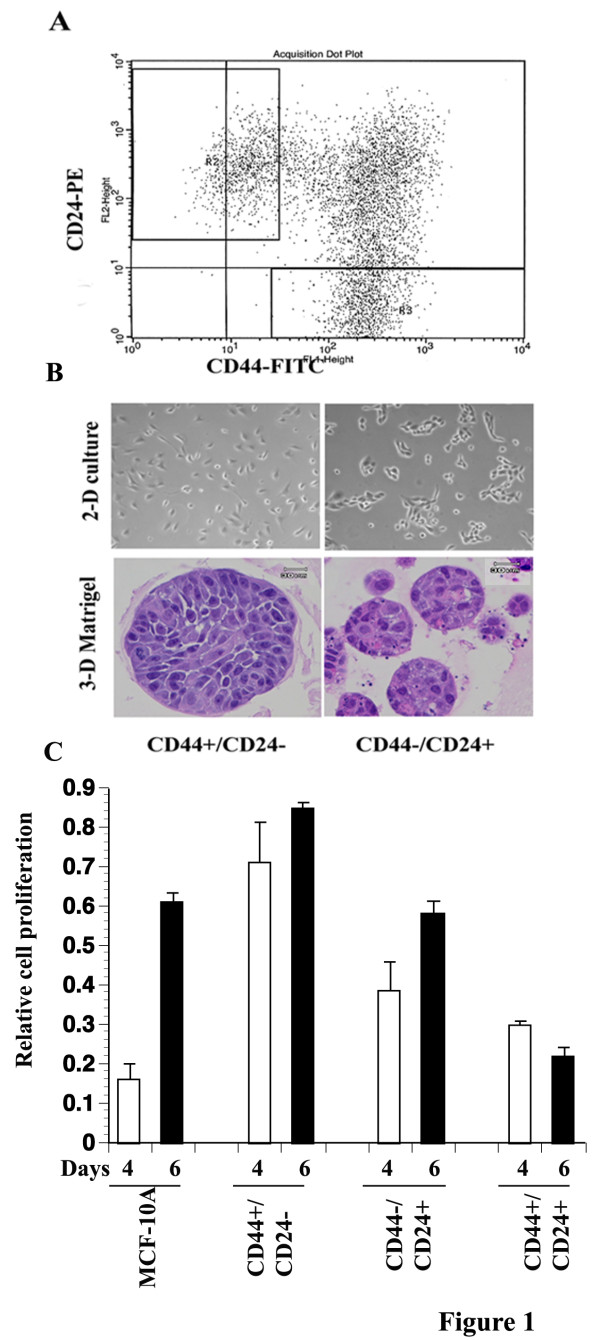
**Growth properties of the CD44+/CD24- and CD44-/CD24+ subpopulations of MCF-10A cells**. A) Flow cytometric analysis of MCF-10A cells labeled with CD44-FITC and CD24-PE antibodies. Total MCF-10A cells can be divided into three distinct subpopulations: CD44+/CD24-, CD44-/CD24+, and CD44+/CD24+. B) Representative growth characteristics of CD44+/CD24- and CD44-/CD24+ cells in 2 D culture (top panel) and 3D-matrigel cultures. Hematoxylin & Eosin staining was performed to characterize cells grown in matrigel. C) Proliferation rate of CD44+/CD24-, CD44-/CD24+, and CD44+/CD24+ cells. Proliferation rate was measured using bromodeoxyuridine incorporation assay. Representative data from two experiments, both done in duplicate with eight wells per cell type, are shown.

MCF-10A cells were originally isolated from fibrocystic disease and are believed to contain stem/basal/myoepithelial cells [39,45,46]. Depending on confluency of plating, a subpopulation of these cells spontaneously acquires CD44+/CD24- phenotype [47]. Three distinct subpopulations could be detected with these cells; CD44+/CD24-, CD44+/CD24+, and CD44-/CD24+ (Figure 1A). Isolated CD44+/CD24- MCF-10A cells displayed a density dependent growth rate: growth was slower than CD44-/CD24+ cells in 100 mm plates (Figure 1B) but faster in 96 well plates compared to unsorted, CD44-/CD24+ or CD44+/CD24+ cells under same growth conditions (Figure 1C). In general, CD44+/CD24+ cells were difficult to grow; therefore, only a limited number of studies were performed with these cells. In matrigel, CD44+/CD24- cells formed larger acini compared to CD44-/CD24+ cells (Figure 1B).

To determine the differential gene expression pattern in CD44+/CD24- and CD44-/CD24+ cells of this immortalized but not transformed cell line we performed microarray analysis. The analysis was performed in quadruplicate. Two thousand thirty five genes were expressed differentially between the two cell types when the expression differential had a p < 0.001 (Welch's t-test), a fraction of at least 0.5 and an absolute expression level difference of ≥ 2-fold (Additional file 2, Table S2). Among them, 1145 genes showed relatively increased expression in CD44+/CD24- cells. For example, the expression of lumican and connexin-43 were higher by more than 350-fold in CD44+/CD24- cells compared to CD44-/CD24+ cells. In contrast, CD44-/CD24+ cells expressed more than 100-fold higher levels of several members of kallikrein family (5, 7 and 10), E-cadherin, and P-cadherin. CD44+/CD24- cells expressed 16-fold lower levels of claudin; breast epithelial cells expressing lower levels of claudin display primitive stem cell characteristics (CD49f^hi^EpCAM-ER-PR-HER2-CD24-CD133^lo^) [20]. Therefore, the CD44+/CD24- subpopulation of MCF-10A cells used in this study may be the stem cell source that primarily maintains the cell line.

The number of genes that we observed to be differentially expressed was significantly higher than the number of differentially expressed genes obtained with purified normal or cancerous CD44+ and CD24+ cells from primary tissues in other studies. For example, with a p value of ≤ 0.05, Shipitsin et al observed differential expression of 1114 and 1207 genes between CD44+ and CD24+ cells of normal breast and cancer, respectively [19]. Liu et al observed differential expression of 186 genes in tumorigenic CD44+/CD24- compared to normal breast epithelium [13]. Furthermore, the number of genes identified in our study is significantly higher than the number of genes identified as differentially expressed in MCF-10A cells undergoing spontaneous EMT due to differences in confluency [47]. These differences could be related to the relative homogeneity of our cells compared to cells obtained from normal breast or breast cancers. Nonetheless, we found a significant overlap in genes that were identified in our study with that of Shipitsin et al [19]. For example, PROCR, which was described as an additional marker of CD44+/CD24- cells, is highly expressed in the CD44+/CD24- fraction of MCF-10A cells. We also found increased expression of interleukin 8 (IL-8) in CD44+/CD24- cells compared to CD44-/CD24+ cells (Table 1). IL-8 has recently been shown to increase the proportion of CSCs with mammosphere forming ability [48].

**Table 1:** EMT-associated genes overexpressed in CD44+/CD24- cells compared to CD44-/CD24+ cells.

Gene Symbol	Gene name	GenBank number	Fold- change
AKT3	V-AKT murine thymoma oncogene homolog 3	NM_005465	2.0
BDNF	Brain-derived neurotropic factor	NM_001709	28.8
CDH2	Cadherin-2 (N-cadherin)	NM_001792	29.8
CTGF	Connective tissue growth factor	NM_001901	33.5
DAB2	Disabled homolog 2	NM_001343	2.4
FGFR1	Fibroblast growth factor receptor 1	NM_023105	2.6
FYN	FYN oncogene related to SRC, FGR, YES	NM_002037	10.1
HMGA2	High mobility AT-hook 2	NM_003483	24.4
IL8	Interleukin 8	NM_000584	4.8
ILK	Integrin-linked kinase	NM_004517	2.1
ITF2/TCF4	Transcription factor 4	NM_003199	2.1
JAG1	Jagged 1	NM_000214	2.6
JAK2	Janus kinase 2	NM_004972	2.0
MAP4K4	Mitogen activated protein kinase kinase kinase kinase	NM_017792	2.0
MMP-2	Matrix metalloproteinase 2	NM_004530	14.5
NR2F1/COUP-TF1	Nuclear receptor subfamily 2, group F, member 1	NM_005654	17.9
Periostin	Periostin, osteoblast specific factor	NM_006475	81.7
PIK3R1	PI3 kinase regulatory subunit (p85)	NM_181523	2.7
PRKC alpha	Protein kinase C alpha	NM_002737	6.8
S100A4	S100, calcium binding protein A4	NM_002961	7.0
SMAD3	SMAD, mother against DPP homolog 3	NM_005902	2.0
SMAD7	SMAD, mother against DPP homolog 7	NM_005904	3.4
SMURF2	SMAD-specific E3 ubiquitin ligase 2	NM_022739	2.1
SNAI2/SLUG	SNAIL homolog 2	NM_003068	2.3
SPARC	Secreted protein cysteine-rich (osteonectin)	NM_003118	107.7
TGFβ1	Transforming growth factor beta 1	NM_011577	4.6
TGFβ2	Transforming growth factor beta 2	NM_003238	3.4
TWIST2	TWIST homology 2	NM_057179	4.8
Wnt5A	Wingless type, MMTV integration family member, type 5A	NM_003392	6.9
Wnt5B	Wingless type, MMTV integration family member, type 5B	NM_030775	60.1
ZEB-1/TCF8	Transcription factor 8	NM_024285	27.8
ZEB-2/ZFHX1B	Zinc finger E-box binding homeobox 2	NM_014795	8.3

### Validation of select differentially expressed genes

**Figure 2:**
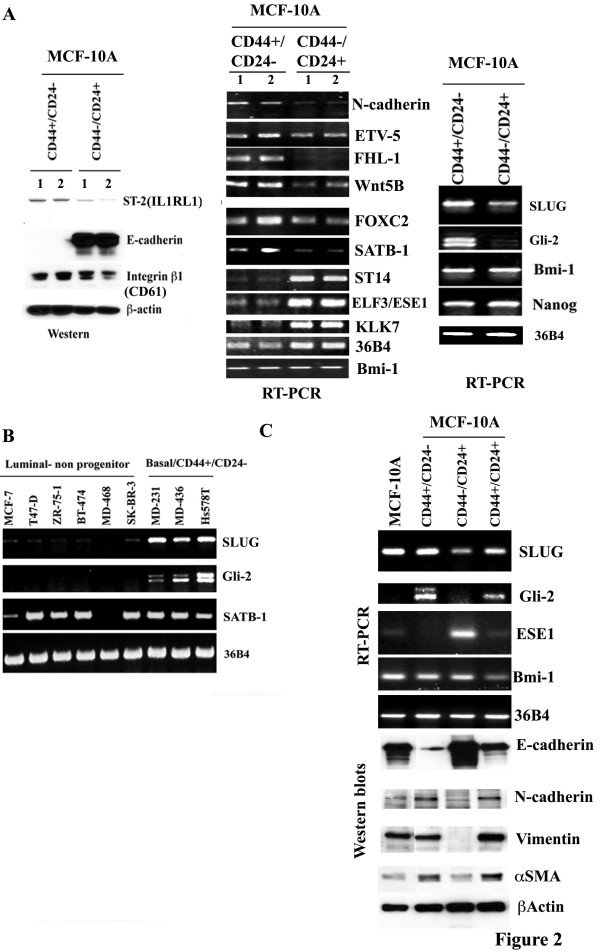
**Western blot and RT-PCR analysis of genes differentially expressed in CD44+/CD24-, CD44-/CD24+ and CD44+/CD24+ cells**. A) CD44+/CD24- cells express higher levels of ST-2, N-cadherin, ETV5, FHL-1, Wnt5B, FOXC2, SATB-1, SLUG, and Gli-2, whereas CD44-/CD24+ cells express higher levels of E-cadherin, ST14, ESE1, and KLK7. Western blotting or RT-PCR was performed to measure protein (left panel) or RNA (central panel) from two independent samples (labeled 1 and 2). B) Cell lines with CD44+/CD24- subpopulation express higher levels of SLUG and Gli-2. C) Expression levels of EMT markers and few additional genes in unsorted MCF-10A, CD44+/CD24-, CD44-/CD24+, and CD44+/CD24+ cells. RT-PCR was used to measure SLUG, Gli-2, ESE1, and Bmi-1, whereas western blotting was used to measure E-cadherin, N-cadherin, vimentin, and αSMA.

To validate differentially expressed genes in different subpopulation of cells, we performed western blot and RT-PCR analysis. Some of the assays were done with two independent samples (indicated as 1 and 2 in Figure 2A). In western blot analysis, we observed elevated expression of ST-2 (IL-1RL1) in CD44+/CD24- cells compared to CD44-/CD24+ cells (Figure 2A, left panel); this gene was elevated in CD44+/CD24- cells by 64-fold at mRNA level (Additional file 2, Table S2). There was a modest increase in integrin beta 1 (CD61) in CD44+/CD24- cells compared to CD44-/CD24+ cells. In contrast, CD44+/CD24- cells expressed very little E-cadherin protein compared to CD44-/CD24+ cells; the difference in E-cadherin (CDH1) expression at the mRNA level between the two cell types was 192-fold (Additional file 2, Table S2).

We confirmed differential expression of several other genes by RT-PCR. For example, the expression of stemness-associated genes was elevated in CD44+/CD24- cells compared to CD44-/CD24+ cells (Figure 2A, central and right panel). Specifically, we observed elevated expression of N-cadherin, FOXC2, ETV5, Wnt5B, and SLUG in CD44+/CD24- cells compared to CD44-/CD24+ cells. In contrast to the above genes, ST14, ESE1, and KLK7, which showed higher expression levels in CD44-/CD24+ cells (> 140-fold) compared to CD44+/CD24- cells in microarray (Additional file 2, Table S2), displayed elevated expression in CD44-/CD24+ cells in RT-PCR assay (Figure 2A, central panel).

Among genes that form a part of the transcription factor network in breast tissue stem cells [16], increased expression of Gli-2 (downstream of Hedgehog) was observed in CD44+/CD24- cells while Bmi-1 and Nanog expression did not differ between CD44+/CD24- and CD44-/CD24+ cells (Figure 2A, right panel). Analysis of several breast cancer cell lines revealed the co-expression of Gli-2 and SLUG in only breast cancer cell lines with CD44+/CD24- phenotype (Figure 2B). SATB-1 has been recently described as a gene responsible for breast cancer metastasis [49]. All SATB-1 expressing cell lines used in that study were enriched for cells with the CD44+/CD24- phenotype [21]. We observed elevated expression of the SATB-1 gene in MCF-10A CD44+/CD24- cells compared to CD44-/CD24+ cells by microarray (Additional file 2, Table S2) and RT-PCR (Figure 2A). However, unlike SLUG and Gli-2, SATB-1 is expressed in majority of cell lines tested and its expression did not correlate with CD44+/CD24- phenotype.

Expression levels of several of the genes noted above were further examined in parental MCF-10A cells and sorted CD44+/CD24-, CD44-/CD24+, and CD44+/CD24+ subpopulations. SLUG and Gli-2 expression was highest in CD44+/CD24- cells followed by CD44+/CD24+ cells (Figure 2C). In contrast, ESE1 expression was highest in CD44-/CD24+ cells. CD44+/CD24- cells and to a lesser extent CD44+/CD24+ cells displayed characteristics of EMT by displaying lower levels of E-cadherin and expressing elevated levels of N-cadherin, vimentin, and αSMA compared to CD44-/CD24+ cells (Figure 2C, bottom panel). These results further support the link between EMT and the CD44+/CD24- phenotype. CD44+/CD24+ cells appear to be of intermediate phenotype expressing moderate levels of E-cadherin but yet expressing N-cadherin and vimentin to a level similar to CD44+/CD24- cells.

### Genes differentially expressed in CD44+/CD24- cells are part of specific signaling networks

We performed Ingenuity pathway analysis to identify signaling networks that may be differentially active in CD44+/CD24- and CD44-/CD24+ cells. Genes that are upregulated in CD44+/CD24- cells are components of five signaling networks: the follicular stimulating hormone (FSH) signaling pathway; TNF/ERK/human chorionic gonadotropin (HCG); p38/IFNα/PKc/JNK; NF-κB/AKT/PDGFβ; and ESR1/BRCA1/CEBPβ pathways (Additional files 3, 4, 5, 6, 7, Figures S1-S5). Genes that were downregulated in CD44+/CD24- cells compared to CD44-/CD24+ cells are components of the FSH/HCG; NF-κB; ERK/p38 kinase; AP-2/GRB2/ZFP36; and ERBB2/TGFβ/RHO pathways (Additional files 8, 9, 10, 11, 12, Figures S6-S10).

**Figure 3:**
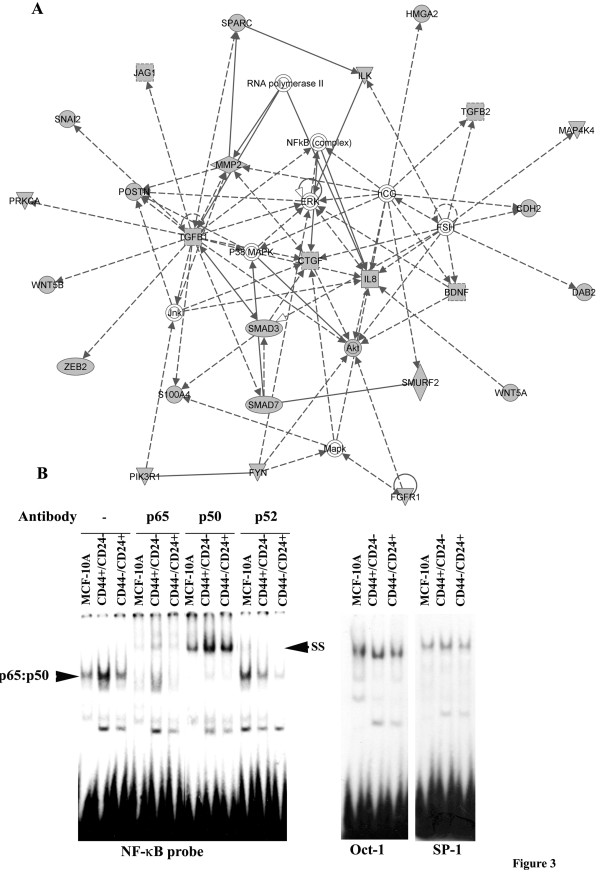
**Signaling network involving CD44+/CD24-enriched EMT-associated genes.** A) Ingenuity pathway analysis of EMT-associated genes upregulated in CD44+/CD24- cells compared to CD44-/CD24+ cells. Genes in shaded shapes correspond to genes that are upregulated in CD44+/CD24- cells compared to CD44-/CD24+ cells. B) NF-κB:DNA complexes in unsorted MCF-10A, CD44+/CD24-, and CD44-/CD24+ subpopulation of cells. CD44+/CD24- cells contained higher levels of p65:p50 heterodimers compared to other cell types. SS = supershift. EMSAs with Oct-1 and SP-1 probes are shown as controls.

Since EMT is linked to the CSC phenotype [37,38], we compiled a list of EMT associated genes that exhibited increased expression in CD44+/CD24- cells. Thirty-two such EMT associated genes were identified (Table 1). This list included most of the well-characterized EMT-associated genes with the exception of SNAIL. Ingenuity pathway analysis identified these genes as participating in a network or networks comprising TGFβ, NF-κB, connective tissue growth factor (CTGF), IL8, HCG, and FSH (Figure 3A). Although a role for TGFβ and IL8 pathways in the CSC phenotype is well known [19,48], the significance of CTGF, HCG, and FSH pathways in the CSC phenotype is unknown.

To confirm the differences in NF-κB signaling pathways between CD44+/CD24, and CD44-/CD24+ cells, we performed electrophoretic mobility shift assays (EMSA) with lysates from these two cell types and unsorted parental cells using a NF-κB oligonucleotide probe. NF-κB exists as an inactive complex in the cytoplasm bound to IκB proteins in unstimulated cells. Stimulation of cells with cytokines such as TNF release NF-κB from IκB to allow DNA binding [50]. CD44+/CD24- cells contained significantly higher levels of NF-κB:DNA complex compared to CD44-/CD24+ cells or unsorted parental cells; antibody supershift assay revealed the presence of p65 and p50 subunits of NF-κB in this complex (Figure 3B).

### CD44-/CD24+ cells acquire a CD44+/CD24- phenotype upon overexpression of activated Ras or treatment with tumor necrosis factor (TNF)

Results of the above analysis combined with the lack of E-cadherin, and the increased expression of EMT-associated genes in CD44+/CD24- MCF-10A cells suggested a role for TNF and Ras in generating CD44+/CD24- cells. TNF and Ras can activate a significant number of the above noted pathways (p38, ERK, and NF-κB in particular) in generating cells with CD44+/CD24- phenotype. Additionally, immune-induced EMT has been shown to generate CSCs [5]. We have recently shown that MCF-10A cells undergo EMT upon exposure to TNF or overexpression of the constitutively active NF-κB subunit p65 [40]. P65 mediated EMT correlated with loss of E-cadherin and elevated expression of ZEB-1 and ZEB-2 [40], both of which are expressed at higher levels in CD44+/CD24- cells (Table 1).

**Figure 4:**
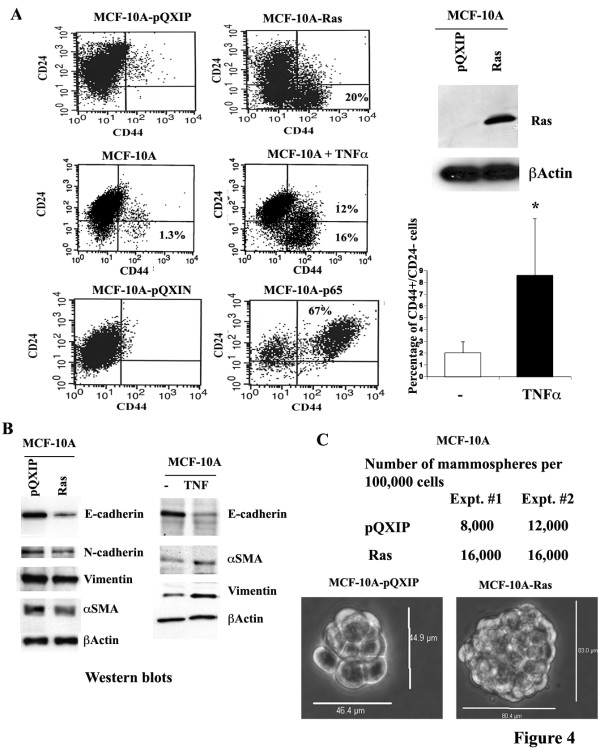
**Ras and TNF induce phenotype associated with cancer stem cells.** A) The effects of Ras or p65 overexpression and TNF treatment on CD44 and CD24 expression status in MCF-10A cells. Activated H-Ras oncogene or the constitutively active p65 subunit of NF-κB was overexpressed by retroviral vector mediated gene transfer. Expression level of Ras in MCF-10A-Ras cells as measured by western blotting is shown (right panel, top) along with number of CD44+/CD24- cells in untreated and TNF treated MCF-10A cells from four experiments (p = 0.03, right bottom panel). MCF-10A-p65 cells have already been described [40]. Cells were treated with 10 ng/ml of TNF for 10 days. B) The effect of Ras overexpression or TNF treatment on the expression of EMT markers in MCF-10A cells. Ras reduced E-cadherin expression, whereas TNF reduced E-cadherin but increased vimentin and αSMA. C) Assay demonstrating the effects of Ras overexpression on mammosphere formation. Cells were plated for 10-days in mammosphere media. Ras increased both the number and the size of mammospheres.

Upon stable transfection of Ras oncogene or exposure to TNF for 10 days, CD44-/CD24+ MCF-10A cells were reanalyzed to assess their CD44 and CD24 phenotype. In both cases, a fraction of cells became CD44+/CD24- (Figure 4A). The effect of Ras in generating CD44+/CD24- cells was accompanied with reduced expression of E-cadherin but not gain of vimentin, similar to a previous report [37] (Figure 4B). The extent of TNF-mediated CD44+/CD24- generation is similar to the previously reported effects of TGFβ1 [37] and is accompanied with reduction in E-cadherin expression and elevated vimentin and αSMA expression. In mammosphere assay, Ras overexpressing cells showed an increase in number as well as size of mammospheres (Figure 4C). However, the effects of TNF on mammosphere formation both on numbers and size were not consistent, possibly due to issues related to half-life or access to mammospheres in semi-solid growth media (data not shown).

P65 altered the sorted cell phenotype from CD44-/CD24+ to CD44+/CD24+ (Figure 4A). The effects of p65 was much more dramatic than the effects of Ras oncogene as more than 65% of cells acquired CD44+/CD24+ phenotype. These results suggest that induced EMT can change the surface expression pattern of CD44 and CD24; however, specific changes are dependent on the inducers of EMT. ZEB-1 and ZEB-2, which are upregulated in MCF-10A cells overexpressing p65 [40], may only change CD44 but not CD24 expression and thus result in a double positive phenotype.

### Overexpression of SLUG, but not Gli-2 or SATB-1, leads to changes in CD44 expression status in breast cancer cell lines

A role for SNAIL and TWIST in the EMT-associated increase in CD44+/CD24- has been demonstrated [38]. However, the possibility that SLUG participates in CD44/CD24 phenotypic changes has not been investigated, although its upregulation in basal breast cancers and mammospheres has been reported recently [51]. Since our observation of increased SLUG expression in CD44+/CD24- cells originated from a cell line study, we first examined the gene expression array data available in NCBI GEO omnibus to correlate SLUG expression with CD44+/CD24- status of primary breast cancers. Previous comparative microarray analysis of CD44+/CD24-/lineage- cancer cells and normal breast epithelial cells revealed specific increased expression of SLUG in the CD44+/CD24- subpopulation, although differences did not reach statistical significance due to small sample size (3 samples for non-CD44 and five samples for CD44+ tumor samples, p = 0.054) [13]. Additionally, recent transcriptome analysis of mammary stem cells, luminal progenitor cells, and mature luminal cells for conserved genes between human and mouse in each of these cell types revealed 3.16 fold elevated SLUG/SNAI2 expression in mammary stem cells compared to progenitor or mature cells [52]. Thus, these independent studies identify SLUG as a Stem/CD44+/CD24- cell associated highly expressed gene. Based on its known role in epigenetic regulation of gene expression through recruitment of histone deacetylase complex [53] and recent studies showing epigenetically controlled transcription factors playing a key role in regulating the mammary epithelial cell phenotype [54], SLUG appeared as an ideal candidate to induce the CD44+/CD24- phenotype. Since SLUG expression correlated with the expression of Gli-2, and SATB-1 is highly expressed in CD44+/CD24- MCF-10A cells, we examined the contribution of these factors in inducing and/or maintaining the CD44+/CD24- phenotype.

**Figure 5:**
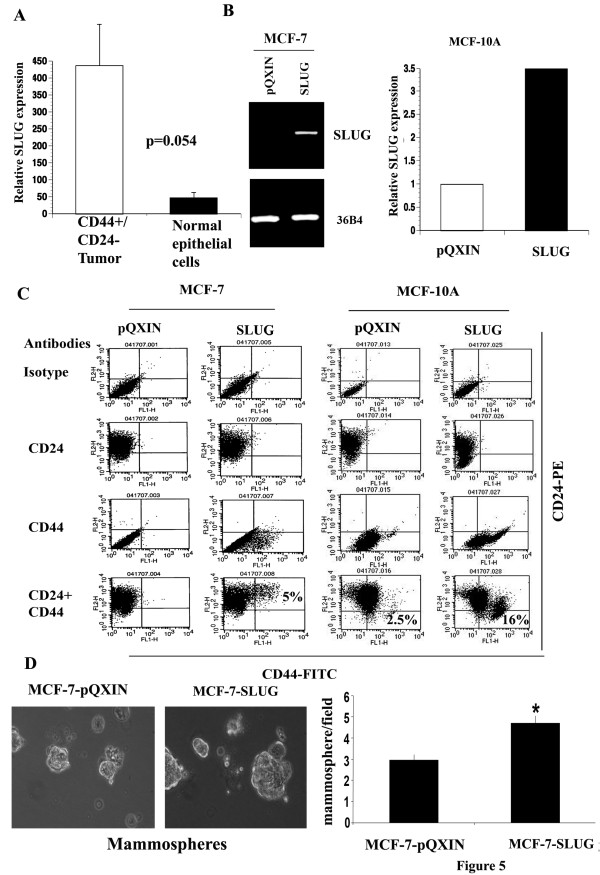
**SLUG overexpression leads to changes in CD44 and CD24 expression pattern**. A) SLUG expression in CD44+/CD24- cells of primary breast cancer compared to normal breast epithelium. These results were generated from the study in Ref 13 with a GEO accession number GDS2617. B) RT-PCR (MCF-7 cells) or qRT-PCR (MCF-10A cells) analysis of overexpressed genes. C) Flow cytometric analysis of different cell types for CD44 and CD24. A subpopulation of MCF-7-SLUG cells acquired the CD44+/CD24+ phenotype, whereas a subpopulation of MCF-10A-SLUG cells acquired the CD44+/CD24- phenotype.

We overexpressed SLUG, Gli-2 and SATB-1 in two cell lines: MCF-10A and MCF-7. Two independent mass cultures of control vector or specific gene overexpressing cells were analyzed. Since both clones had a similar phenotype, results of one clone are presented in each case. MCF-7 is an ERα-positive breast cancer cell line of luminal subtype. For unknown reason, we were unable to generate MCF-10A cells overexpressing SATB-1. The expression levels of SLUG in control vector and SLUG overexpressing cells are shown in Figure 5B. A subpopulation of SLUG overexpressing MCF-10A cells displayed the CD44+/CD24- phenotype (Figure 5C). Although we used a bicistronic retrovirus and cells were selected using a selectable marker, not all CD44-/CD24+ cells acquired CD44+/CD24- phenotype. This suggests a more dominant pathway may be involved in maintaining the CD44-/CD24+ phenotype in MCF-10A cells. Alternatively, overexpression of SLUG may drive the acquisition of the CD44+/CD24- phenotype in a dose dependent manner, or only in a subpopulation of CD44-/CD24+ MCF-10A cells.

A subpopulation of MCF-7-SLUG cells acquired the CD44+/CD24+ phenotype suggesting different effects of SLUG on CD44 and CD24 expression in luminal and basal cell types (Figure 5C). Although CD44+/CD24- cells were originally described as CSCs, in the same original study, CD44+/CD24+ cancer cells isolated from one patient with comedo (more aggressive) type adenocarcinoma were tumorigenic [10]. Therefore, it is possible that CSCs from luminal cell types may be different than those from basal cell types and may be characterized as CD44+/CD24+.

**Figure 6:**
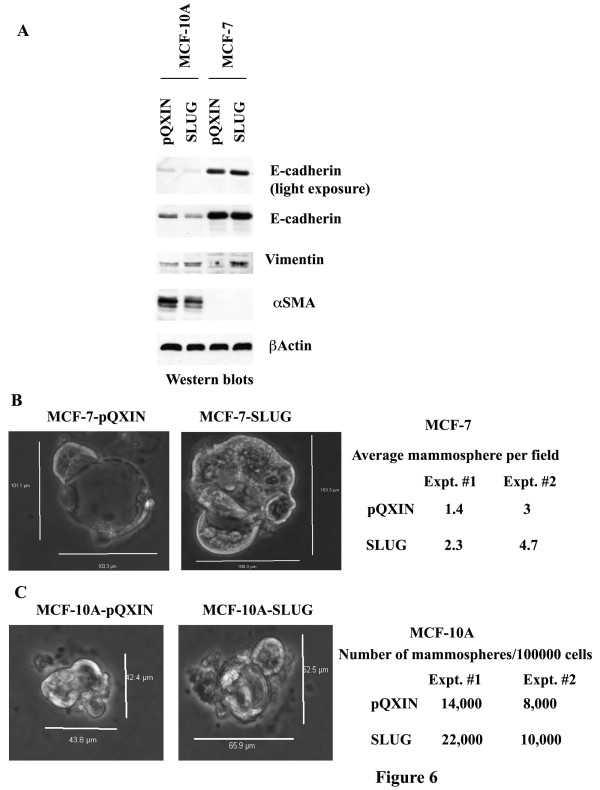
**The effect of SLUG overexpression on the expression of EMT markers**. MCF-10A-SLUG cells showed lower levels of E-cadherin and higher levels of vimentin compared to MCF-10A-pQXIN cells. MCF-7-SLUG cells showed elevated vimentin compared to MCF-7-pQXIN cells. B) Mammosphere forming ability of MCF-7pQXIN, MCF-7-SLUG, MCF-10A-pQXIN, and MCF-10A-SLUG cells. Mammospheres are shown on left, whereas quantitative differences in number of mammospheres formed in two experiments are shown on right panel.

We examined whether changes in CD44 and CD24 expression in SLUG overexpressing cells correlated with altered expression of EMT-associated genes. MCF-10A-SLUG cells displayed lower levels of E-cadherin but elevated vimentin compared to control vector-containing MCF10A-pQXIN cells (Figure 6A). In contrast, MCF-7-SLUG cells showed no change in E-cadherin expression but expressed vimentin compared to MCF-7-pQXIN cells. Thus, SLUG overexpression has cell type-specific effects on the expression of EMT-associated genes. In a mammosphere assay, MCF-7-SLUG cells formed more and larger mammospheres compared to MCF-7-pQXIN cells, suggesting SLUG may confer a CSC phenotype to luminal breast cancer cells and these putative CSCs are phenotypically CD44+/CD24+ (Figure 6B). MCF-10A-SLUG cells appear to form larger mammospheres compared to MCF-10A-pQXIN cells; however, both cell types formed similar number of mammospheres (Figure 6C).

In contrast to SLUG, Gli-2 overexpression did not alter CD44 and CD24 status of either MCF-10A or MCF-7 cells (Additional file 13, Figure S11A for the figure and Additional file 14 for the legend). Although MCF-10A cells used for overexpression of Gli-2 were initially sorted for CD44-/CD24+ cells, due to lower efficiency of plasmid based transfection of Gli-2, selection of transfected cells required longer time compared to retroviral vector-based transduction of SLUG. Therefore, some of the cells have spontaneously drifted to a CD44+/CD24+ phenotype, which was similar between pcDNA3 and Gli-2 transfected cells. MCF-7pcDNA3 and MCF-7-Gli-2 cells formed a similar number of mammospheres; the size of mammospheres was also similar (Additional file 13, Figure S11B). In addition, Gli-2 overexpression in MCF-7 cells did not alter the expression levels of E-cadherin, vimentin or α SMA (Additional file 13, Figure S11C). Similar to Gli-2, SATB-1 did not change the CD44 and CD24 status of MCF-7 cells (Additional file 13, Figure S11D). Therefore, it appears that only SLUG is capable of inducing the CD44+/CD24- phenotype in MCF-10A cells and the CD44+/CD24+ phenotype in MCF-7 cells. However, we cannot rule out that the different transgene delivery method have played a part in the lack of effect from Gli-2.

## Discussion

In this report, we describe gene expression patterns in the CD44+/CD24- and CD44-/CD24+ subpopulations of MCF-10A cells. Unlike previous studies using similar subpopulations of epithelial cells from normal or cancerous breast, our study identified a large number of genes that are differentially expressed between cells with these two phenotypes. This could be partly due to MCF-10A cells being more homogenous, as well as not having to amplify RNA before microarray analysis. Based on the claudin expression pattern, CD44+/CD24- cells that were used in this study appear to more closely resemble stem cells [20]. Several of the genes upregulated 10-fold or higher in CD44+/CD24- cells were cell surface molecules, which can potentially be used to further fractionate CD44+/CD24- cells into distinct subpopulations and assayed for stem/progenitor status. These molecules/markers include EPHA4, EPHA5, ST-2, platelet growth factor receptor-like-1, LRP-1/CD91, and Toll-like receptor 4.

Previous studies linking EMT to the CD44+/CD24- phenotype identified only a few genes that may be involved in generating CD44+/CD24- cells. These genes included TWIST1, TWIST2, SNAIL, SLUG/SNAI2, FOXC2, and ZFHX1B/ZEB-2 [38]. TWIST2, SLUG, and ZEB-2 were also identified in our study to be elevated in CD44+/CD24- cells (Table 1). We observed enhanced expression in CD44+/CD24- cells of an additional 29 genes that have been associated with EMT. Pathway analysis suggested that expression and/or activity of 27 of these genes are linked (Figure 3A). However, not all genes involved in EMT are likely to have the ability to induce the CD44+/CD24- phenotype. We had previously demonstrated that MCF-10A cells overexpressing constitutively active p65 undergo EMT, which is accompanied by upregulation of ZEB-1 and ZEB-2 [40]. However, MCF-10A cells overexpressing p65 displayed a CD44+/CD24+ phenotype, suggesting that ZEB-1 and ZEB-2-mediated EMT did not involve generation of CD44+/CD24- cells. Nonetheless, p65 overexpressing CD44+/CD24+ cells may have stem cell like properties similar to MCF-7-SLUG cells. In this respect, it has recently been demonstrated that SRC-mediated transformation of MCF-10A cells involves the NF-κB-Lin28B-let7-IL-6 axis and this signaling axis generates cancer cells with stem cell like properties [55]. Thus, it is likely that cells with the CD44+/CD24- and CD44+/CD24+ phenotype have stem cell like properties at least based on the mammosphere assay. Clinical prognostic studies using CD44 and CD24 as markers may have to consider both cell types. Since MCF-10A cells that have acquired an EMT phenotype without co-expressing an oncogene such as SRC and Ras or the cytokine IL-6 are not tumorigenic [55], we did not characterize MCF-10A-SLUG or MCF-10A-p65 cells in xenograft models for CSC behavior.

Although SLUG was thought to function in a redundant manner with SNAIL, several recent studies suggest unique functions for SLUG. For the following reasons SLUG appears to be much more relevant for generating breast cancer cells with a CSC phenotype than SNAIL: 1) SLUG is downstream of Notch1 and Jagged 1 and is essential for Notch1:Jagged 1 mediated EMT, tumor growth, and metastasis [56]; 2) Jagged 1 expression in primary breast cancer correlates with SLUG but not SNAIL expression [56]; 3) SLUG expression correlates with poor breast cancer prognosis [57]; 4) SLUG but not SNAIL or TWIST is expressed in ES cells and is part of the ES cell signature activated in several cancer cells [58,59]; 5) SLUG contributes to the function of the stem cell factor c-kit signaling pathways [60]; c-kit is overexpressed in basal breast cancers, which are also predominantly of CD44+/CD24- phenotype [21,61]; 6) E-cadherin, whose experimental depletion in mammary epithelial cells leads to generation of CSC-like cells [62], represses SLUG but not SNAIL or TWIST expression [56]; 7) SLUG is a potent repressor of the tumor suppressor p53 and deregulation of p53 activity is often observed in breast cancers [63]; 8) SLUG-/- embryonic fibroblasts show reduced expression of several genes linked to self-renewal and chromatin remodeling [64]; 9) SLUG confers resistance to radiation, a characteristic of CD44+/CD24- cells [65,66]; 10) SLUG but not SNAIL expression, is linked to ductal development in the breast including tubule maintenance or growth within invasive ductal carcinoma [67]; and 11) SLUG is highly expressed in basal type breast cancers, which also tend to express higher levels of stem cell-associated genes [20,51,68]. These observations along with the results of this study highlight critical role of SLUG in breast cancer.

Gli-2, a hedgehog pathway mediator linked to EMT [69], failed to alter the CD44 and CD24 phenotype of MCF-10A cells. This is somewhat surprising considering previous reports showing elevated expression of Gli-2 in primary mammary stem/progenitor cells and its association with increased mammosphere-forming ability [16]. Overall our results raise two important issues: 1) not all EMT associated genes can induce the CD44+/CD24- phenotype when overexpressed; and 2) not all breast epithelial subtypes (luminal versus basal) acquire a subpopulation of cells with the CD44+/CD24- phenotype upon overexpression of EMT-associated genes. In this respect, our results agree with the observation that primary breast cancers expressing EMT markers are predominantly of basal-like phenotype [47,51].

The ability of only a few EMT-associated genes to induce the EMT phenotype and upregulate the expression of genes linked to the CD44+/CD24- phenotype suggests that genes with this dual capacity have stronger transcriptional activity, and in particular target chromatin modifying genes. Several chromatin modifying genes including HMGA2, EPC2, CBX6, SMARCA1, and SMARCA3 showed increased expression in CD44+/CD24- cells (Additional file 2, Table S2). Future studies focusing on these genes may help to identify drugs that target the CD44+/CD24- subpopulation of cancer cells, as has been demonstrated recently using human mammary epithelial cells depleted of E-cadherin or overexpressing TWIST [62].

## Conclusions

In this study, we have demonstrated increased expression of 32 EMT- associated genes in breast epithelial cells with a CD44+/CD24- phenotype compared to CD44-/CD24+ phenotype. Among these genes, SLUG was able to convert CD44-/CD24+ cells to CD44+/CD24- or CD44+/CD24+ cells, depending upon the cell line. Treatment with TNF also could induce the CD44+/CD24- phenotype, further supporting a role for the microenvironment in generating cells with stem cell phenotype.

## List of abbreviations

ALDH1: Aldehyde dehydrogenase 1; αSMA: Alpha Smooth Muscle Actin; CSC: Cancer stem cells; CTGF: Connective tissue growth factor; CDH1: E-cadherin; EMSA Electrophoretic mobility shift assay; EMT: Epithelial to mesenchymal transition; FSH: Follicular stimulating hormone; GEO: Gene Expression Omnibus; HGF: Hepatocyte growth factor; MAS5: MicroArray Suite 5; PDGF: Platelet derived growth factor: RT-PCR: Reverse transcription polymerase chain reaction; SIP1; Smad-Interacting Protein; TGFβ: Transforming growth factor beta; TNF: Tumor necrosis factor.

## Competing interests

HN, SB and Indiana University have submitted a patent application for ANTXR1, which is expressed at higher levels in CD44+/CD24- cells, as a marker and target for breast cancer stem cells.

## Authors' contributions

PBN performed most of the experiments described in the manuscript including generating cell lines and RT-PCR. HA did mammosphere assays and RT-PCR. CB and EFS designed and performed several flow cytometry assays. PF did RT-PCR studies, whereas SB did H&E analysis of 3 D cultures and writing the manuscript. RG provided primary tumor samples, which were analyzed as part of this study and participated in the design of the study. HN was responsible for designing experiments, flow cytometry, and writing the manuscript. All authors have read and approved the final manuscript.

## Pre-publication history

The pre-publication history for this paper can be accessed here:

http://www.biomedcentral.com/1471-2407/10/411/prepub

## Supplementary Material

Additional file 1 **Table 1: List of primers used for RT-PCR**. This file contains sequence of primers used for polymerase chain reactions. Click here for file

Additional file 2 **Table 2: List of genes differentially expressed in CD44+/CD24- and CD44-/CD24+ subpopulation of MCF-10A cells**. This files contains list of genes that showed statistically significant difference in expression (p < 0.001, ≥ 2-fold change) between CD44+/CD24- and CD44-/CD24+ cells. Fold change in negative value corresponds to genes expressed at higher levels in CD44+/CD24- cells and in positive values corresponds to genes expressed at higher levels in CD44-/CD24+ cells. Click here for file

Additional file 3 **Figure S1: Ingenuity pathway analysis of genes expressed at higher levels in CD44+/CD24- cells**. The majority of genes in this pathway are linked to signaling by follicular stimulating hormone (FSH). Shaded symbols indicate genes that are expressed at higher levels. Click here for file

Additional file 4 **Figure S2: Ingenuity pathway analysis of genes expressed at higher levels in CD44+/CD24- cells**. The second signaling network links highly expressed genes in CD44+/CD24- cells to ERK and human chorionic gonadotropin (hCG) signaling. Click here for file

Additional file 5 **Figure S3: Ingenuity pathway analysis of genes expressed at higher levels in CD44+/CD24- cells**. The third signaling network links highly expressed genes in CD44+/CD24- cells to p38, JNK and interferon alpha. Click here for file

Additional file 6 **Figure S4: Ingenuity pathway analysis of genes expressed at higher levels in CD44+/CD24- cells**. The fourth signaling pathway links highly expressed genes in CD44+/CD24- cells to NF-κB. Click here for file

Additional file 7 **Figure S5: Ingenuity pathway analysis of genes expressed at higher levels in CD44+/CD24- cells**. The fifth signaling pathway links highly expressed genes in CD44+/CD24- cells to BRCA1 and estrogen receptor (ESR1). Click here for file

Additional file 8 **Figure S6: Ingenuity pathway analysis of genes expressed at lower levels in CD44+/CD24- cells compared to CD44-/CD24+ cells**. Genes in this network are linked to hCG and FSH Click here for file

Additional file 9 **Figure S7: Ingenuity pathway analysis of genes expressed at lower levels in CD44+/CD24- cells compared to CD44-/CD24+ cells**. Genes in this network are linked to NF-κB and IL-12 Click here for file

Additional file 10 **Figure S8: Ingenuity pathway analysis of genes expressed at lower levels in CD44+/CD24- cells compared to CD44-/CD24+ cells**. Genes in this network are linked to PI3 kinase/AKT Click here for file

Additional file 11 **Figure S9: Ingenuity pathway analysis of genes expressed at lower levels in CD44+/CD24- cells compared to CD44-/CD24+ cells**. Genes in this network are linked to multiple signaling molecules including growth hormone (GH1) and GRB2. Click here for file

Additional file 12 **Figure S10: Ingenuity pathway analysis of genes expressed at lower levels in CD44+/CD24- cells compared to CD44-/CD24+ cells**. Genes in this network are linked to ERBB2 and TGFβ1. Click here for file

Additional file 13 **Figure S11: The effect of Gli-2 and SATB1 overexpression on CD44 and CD24 cell surface expression**. This figure shows the inability of Gli-2 and SATB-1 to alter cell surface CD44 and CD24 expression profile, mammosphere formation, and EMT-associated gene expression. Click here for file

Additional file 14 **Legend for figures and table in additional files**. This file provides detailed legend for all figures and tables, particularly Figure S11. Click here for file
