# Supplementary material for: SLUG/SNAI2 and Tumor Necrosis Factor Generate Breast Cells With CD44+/CD24- Phenotype
Source: BMC Cancer. 2010 Aug 6;10:411. doi: 10.1186/1471-2407-10-411 (PMC3087321; doi:10.1186/1471-2407-10-411)
Supplement: Additional file 1 — Table 1: List of primers used for RT-PCR. This file contains sequence of primers used for polymerase chain reactions. [file 1471-2407-10-411-S1.DOC]

**Table S1:** Sequences of primers used for RT-PCR and/or qRT-PCR

| Gene Name | Forward | Reverse |
| --- | --- | --- |
| 36B4 | TCGACAATGGCAGCATCTAC | GCCTTGACCTTTTCAGCAAG |
| ESE1 | CTGAGCAACCCCAGATGTC | ACCAGACGCAGCTCCTCAAG |
| FOXC2 | TGAAGCCGCCCTACAGCTAC | GCACTCGTTGAGCGAGAGGT |
| WNT5B | CTTCGCCAAGGAGTTTGTGG | CGCGCTGTCGTACTTCTCCT |
| SLUG | TTTCTGGGCTGGCCAAACATAAGC | ACACAAGGTAATGTGTGGGTCCGA |
| GLI2 | CACCAACCAGAACAAGCAGA | ACCTCAGCCTCCTGCTTACA |
| BMI-1 | CCAGGGCTTTTCAAAAATGA | CCGATCCAATCTGTTCTGGT |
| NANOG | CAAAGGCAAACAACCCACTT | TCTGCTGGAGGCTGAGGTAT |
| FHL-1 | AGCAAGTCATCGGGACTGGA | GTCCTCCACAGCGGTGAAAC |
| ST14 | AGGCTCAAGCGCATCATCTC | CGGATCTCACCCTTTTGCAG |
| SATB-1 | AAGCCTTTGGAGCAACAGGTTTCG | ATTCGGTCTCTTTCAGCTTCCGGT |
| NOTCH3 | TGTGGACGAGTGCTCTATCG | AATGTCCACCTCGCAATAGG |
| ETV5 | CCAAGATCAAACGGCAGCTG | GCCTGAGGTGGGGGAAATAC |
| KLK7 | AAACGCCTGCAATGGTGACT | TGGCGTTAGCGATGCTTTTT |
| HMGA2 | CAACAGTCCCTCTAAAGCAGCTCA | TTCGGCAGACTCTTGTGAGGATGT |
