# Supplementary figures and images for: SLUG/SNAI2 and Tumor Necrosis Factor Generate Breast Cells With CD44+/CD24- Phenotype
Source: BMC Cancer. 2010 Aug 6;10:411. doi: 10.1186/1471-2407-10-411 (PMC3087321; doi:10.1186/1471-2407-10-411)

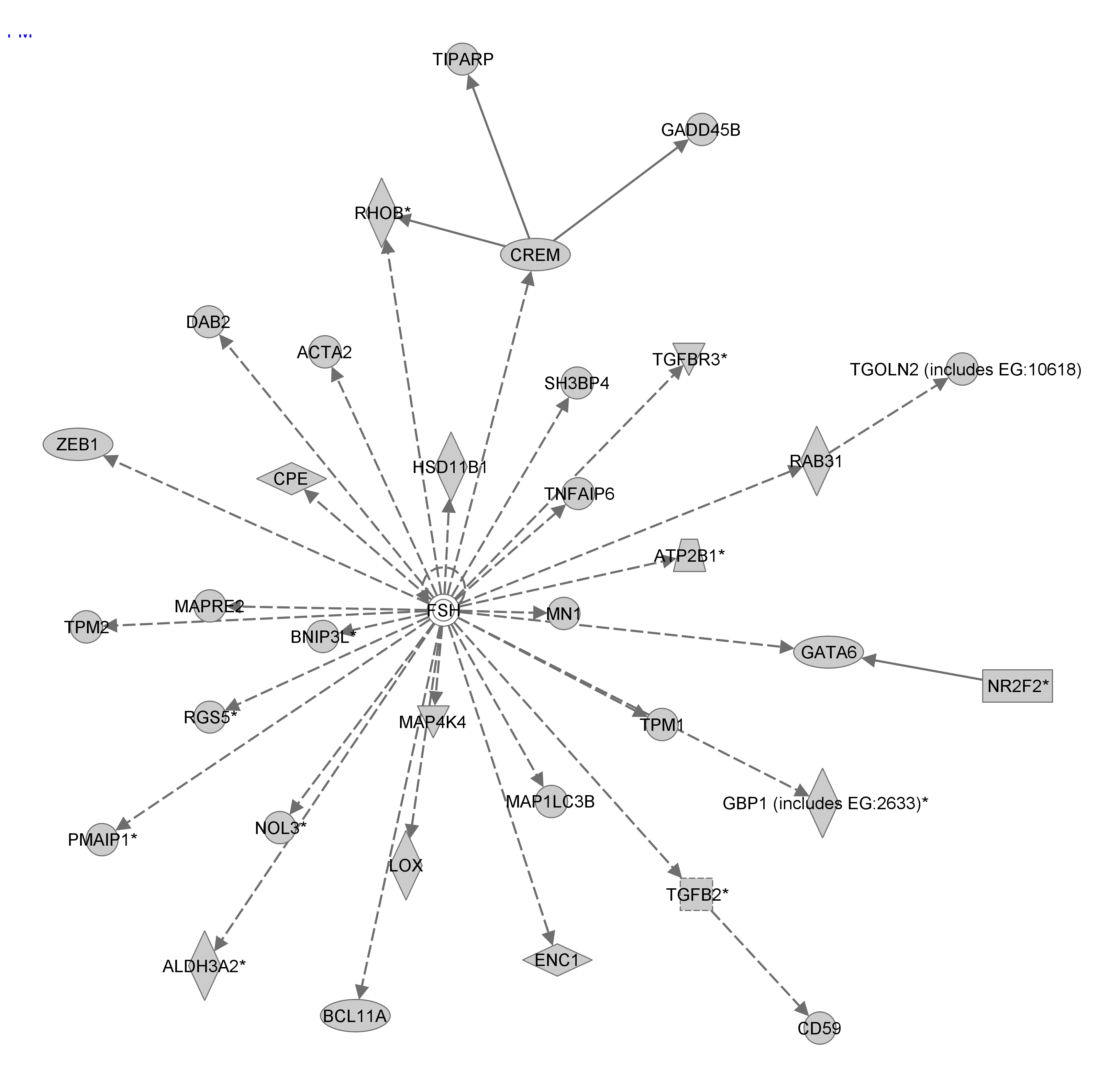

Supplement: Additional file 3 — Figure S1: Ingenuity pathway analysis of genes expressed at higher levels in CD44+/CD24- cells. The majority of genes in this pathway are linked to signaling by follicular stimulating hormone (FSH). Shaded symbols indicate genes that are expressed at higher levels. [file 1471-2407-10-411-S3.JPEG]

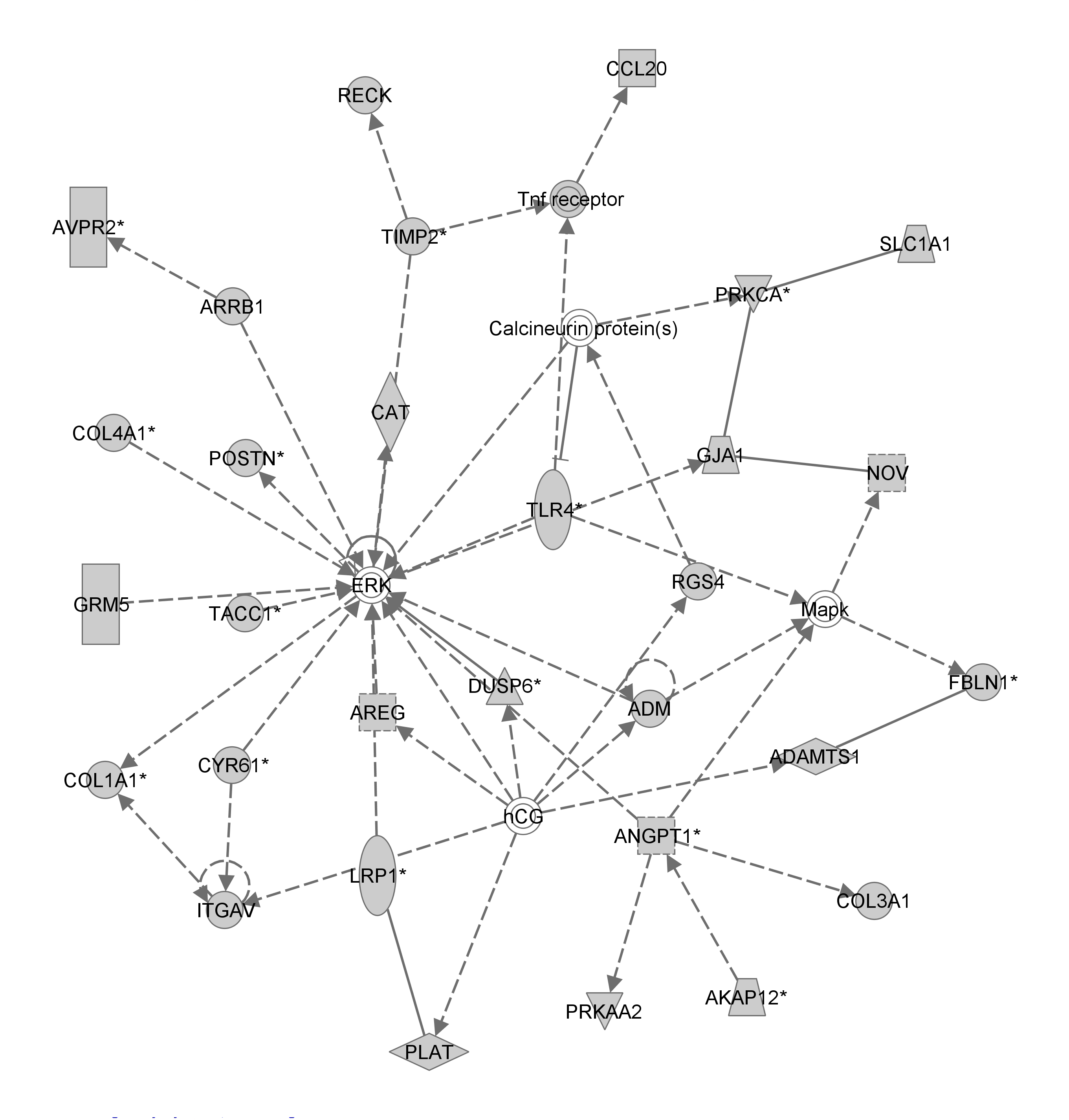

Supplement: Additional file 4 — Figure S2: Ingenuity pathway analysis of genes expressed at higher levels in CD44+/CD24- cells. The second signaling network links highly expressed genes in CD44+/CD24- cells to ERK and human chorionic gonadotropin (hCG) signaling. [file 1471-2407-10-411-S4.JPEG]

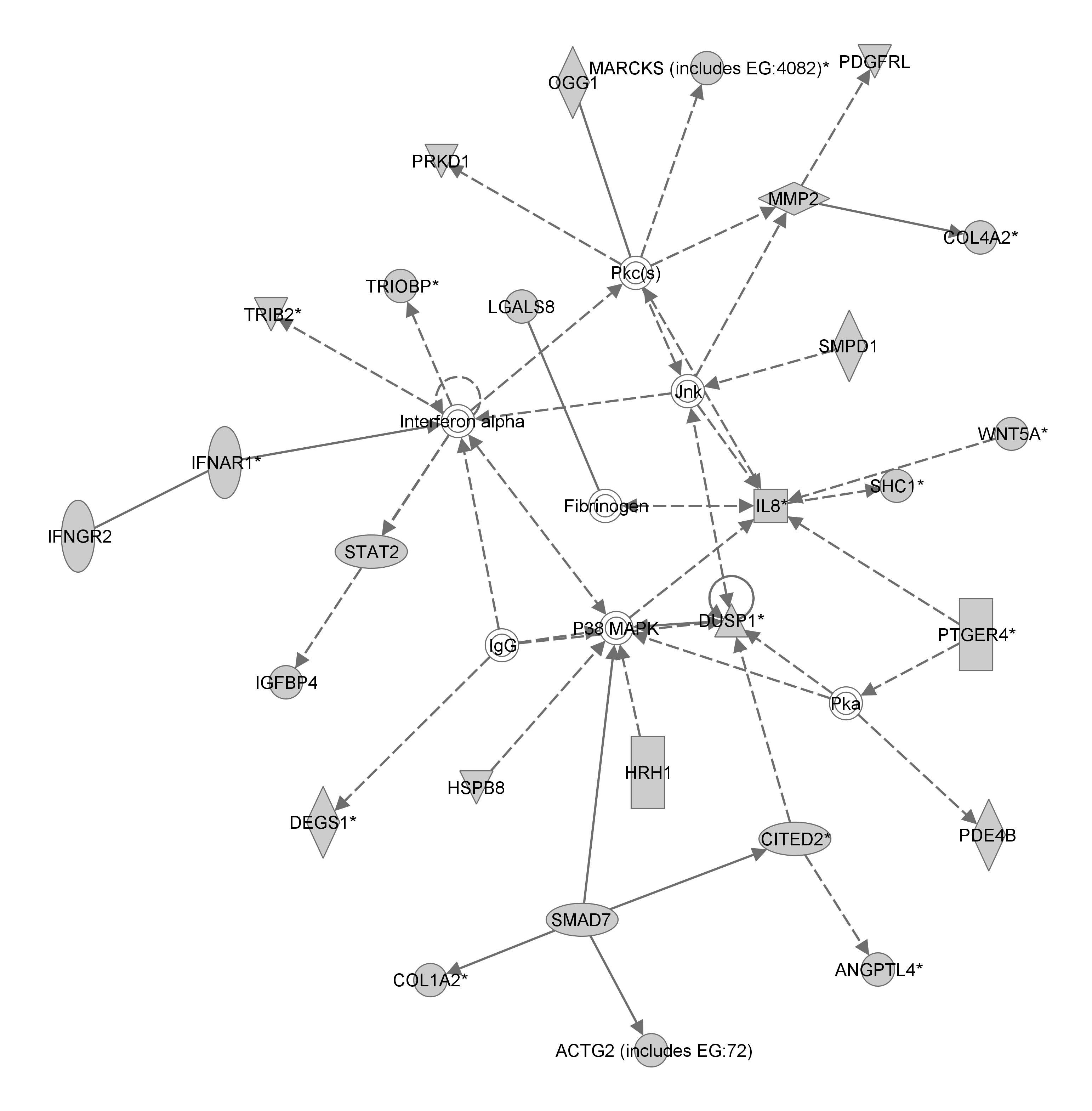

Supplement: Additional file 5 — Figure S3: Ingenuity pathway analysis of genes expressed at higher levels in CD44+/CD24- cells. The third signaling network links highly expressed genes in CD44+/CD24- cells to p38, JNK and interferon alpha. [file 1471-2407-10-411-S5.JPEG]

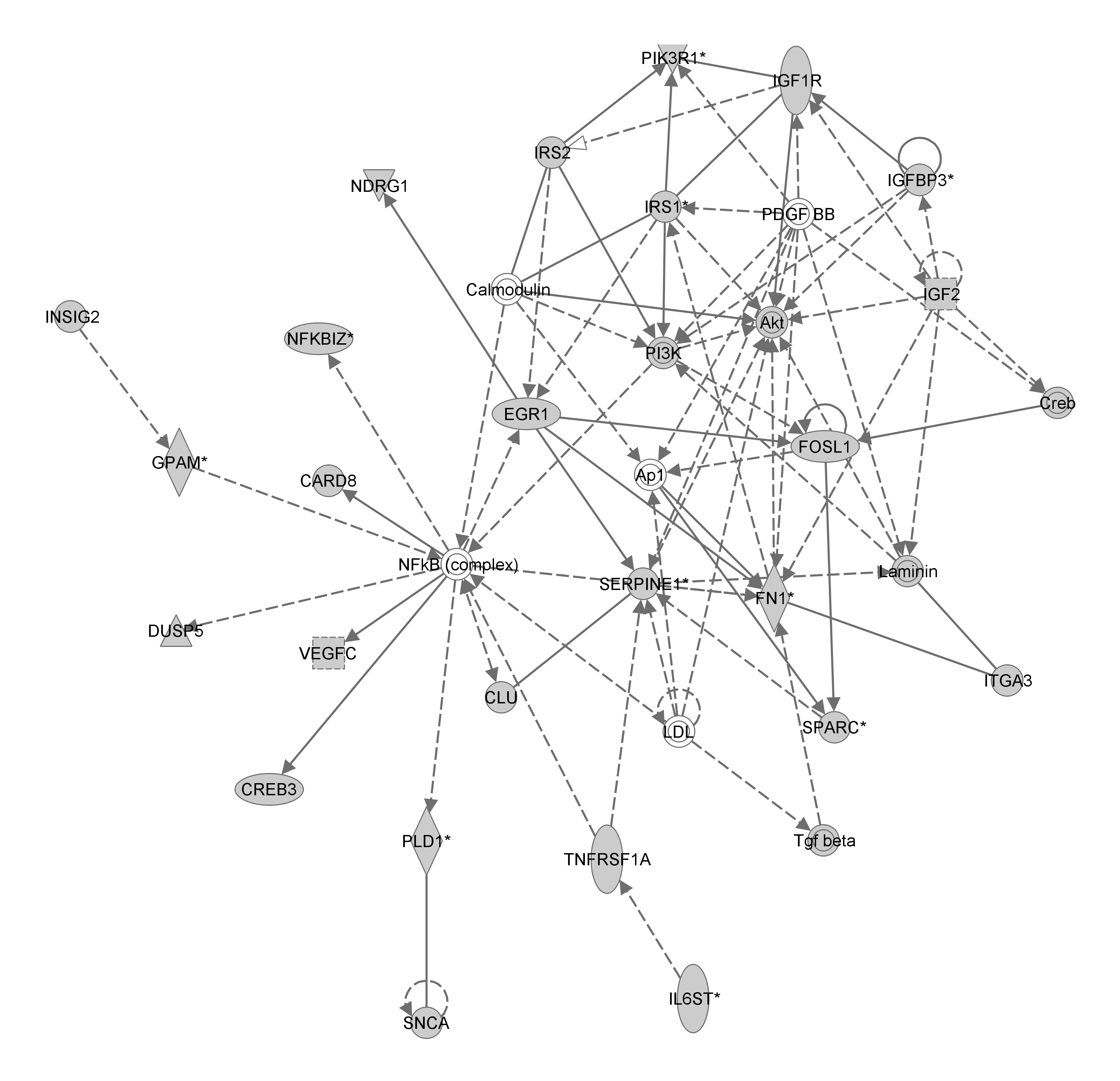

Supplement: Additional file 6 — Figure S4: Ingenuity pathway analysis of genes expressed at higher levels in CD44+/CD24- cells. The fourth signaling pathway links highly expressed genes in CD44+/CD24- cells to NF-κB. [file 1471-2407-10-411-S6.JPEG]

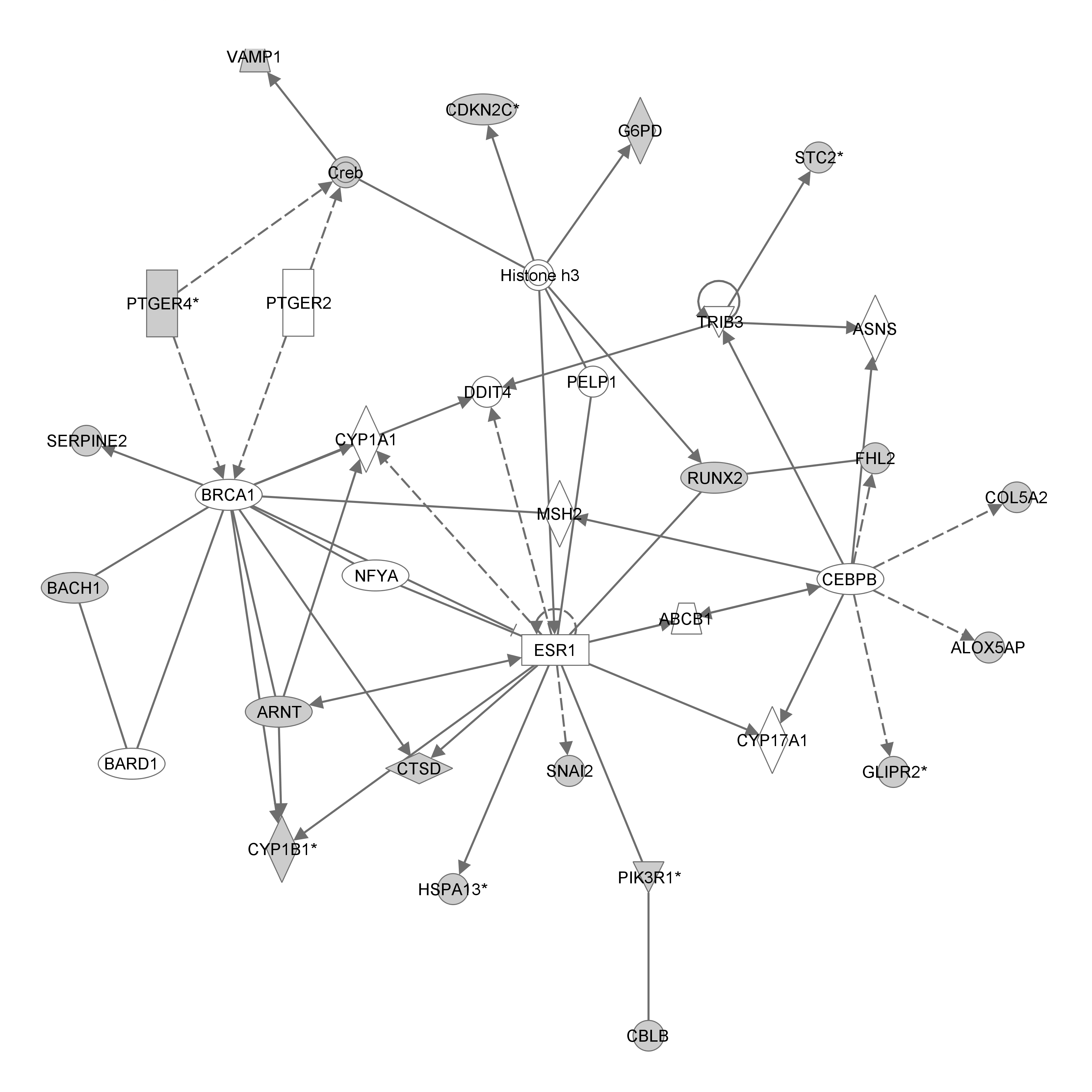

Supplement: Additional file 7 — Figure S5: Ingenuity pathway analysis of genes expressed at higher levels in CD44+/CD24- cells. The fifth signaling pathway links highly expressed genes in CD44+/CD24- cells to BRCA1 and estrogen receptor (ESR1). [file 1471-2407-10-411-S7.JPEG]

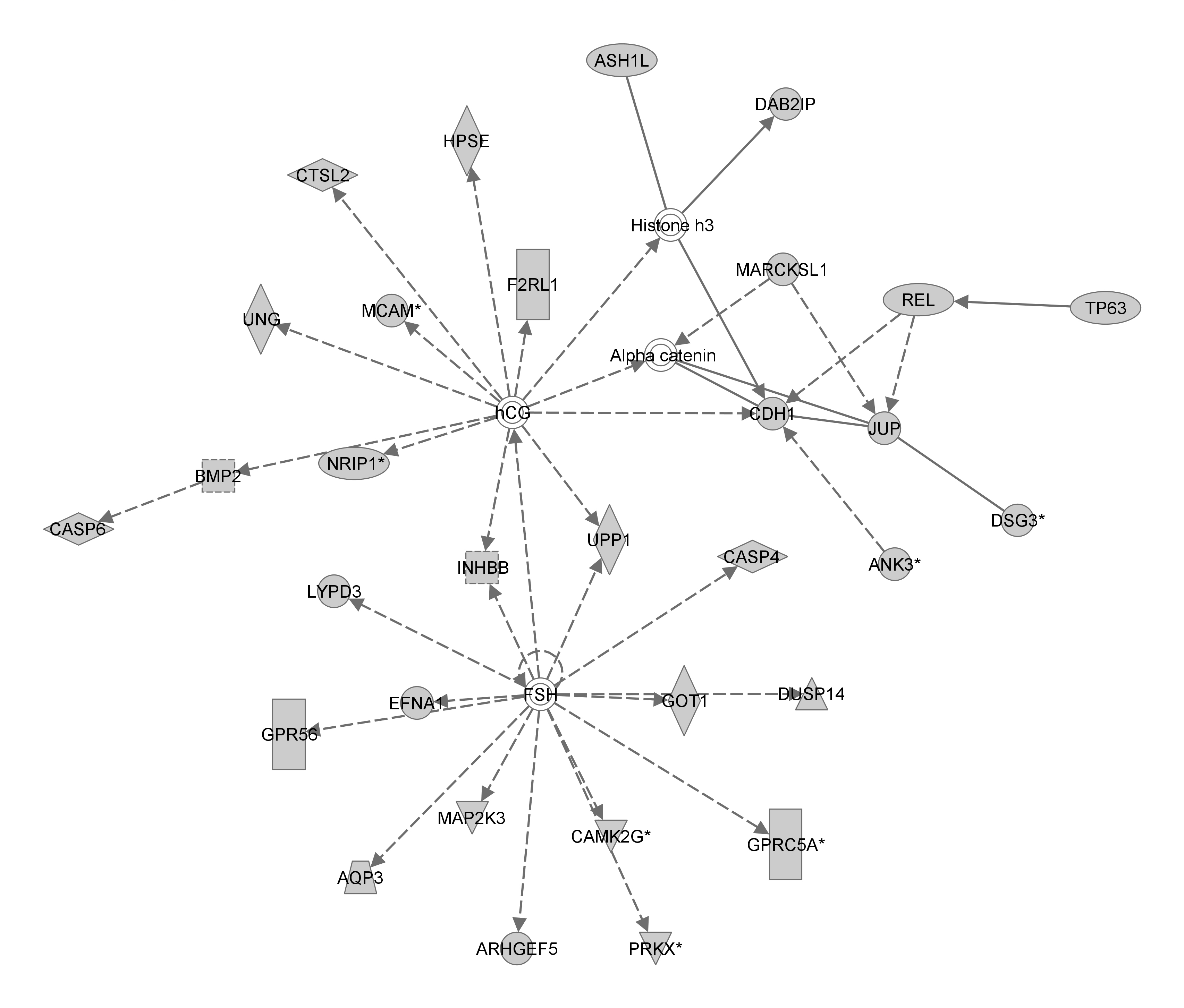

Supplement: Additional file 8 — Figure S6: Ingenuity pathway analysis of genes expressed at lower levels in CD44+/CD24- cells compared to CD44-/CD24+ cells. Genes in this network are linked to hCG and FSH [file 1471-2407-10-411-S8.JPEG]

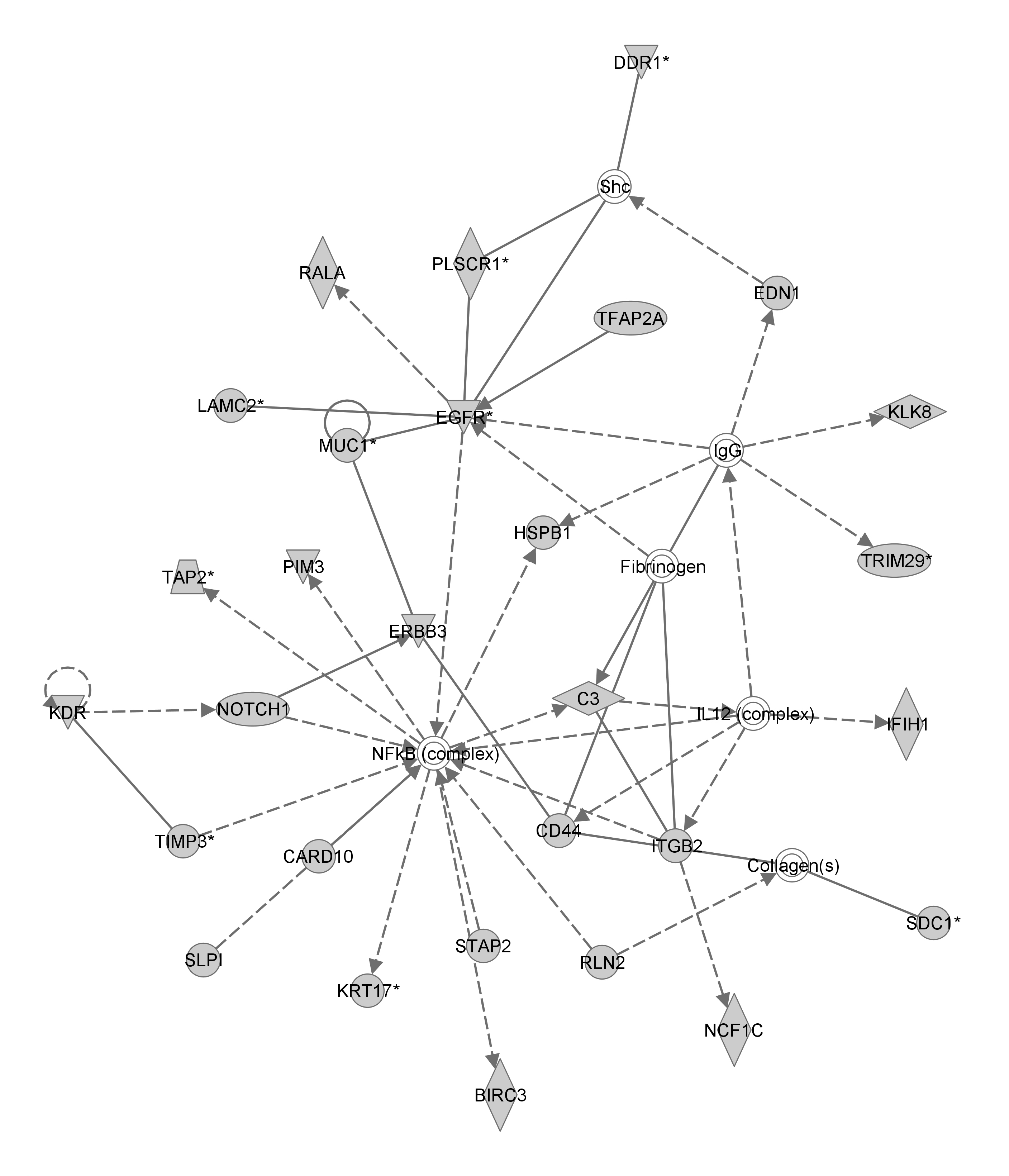

Supplement: Additional file 9 — Figure S7: Ingenuity pathway analysis of genes expressed at lower levels in CD44+/CD24- cells compared to CD44-/CD24+ cells. Genes in this network are linked to NF-κB and IL-12 [file 1471-2407-10-411-S9.JPEG]

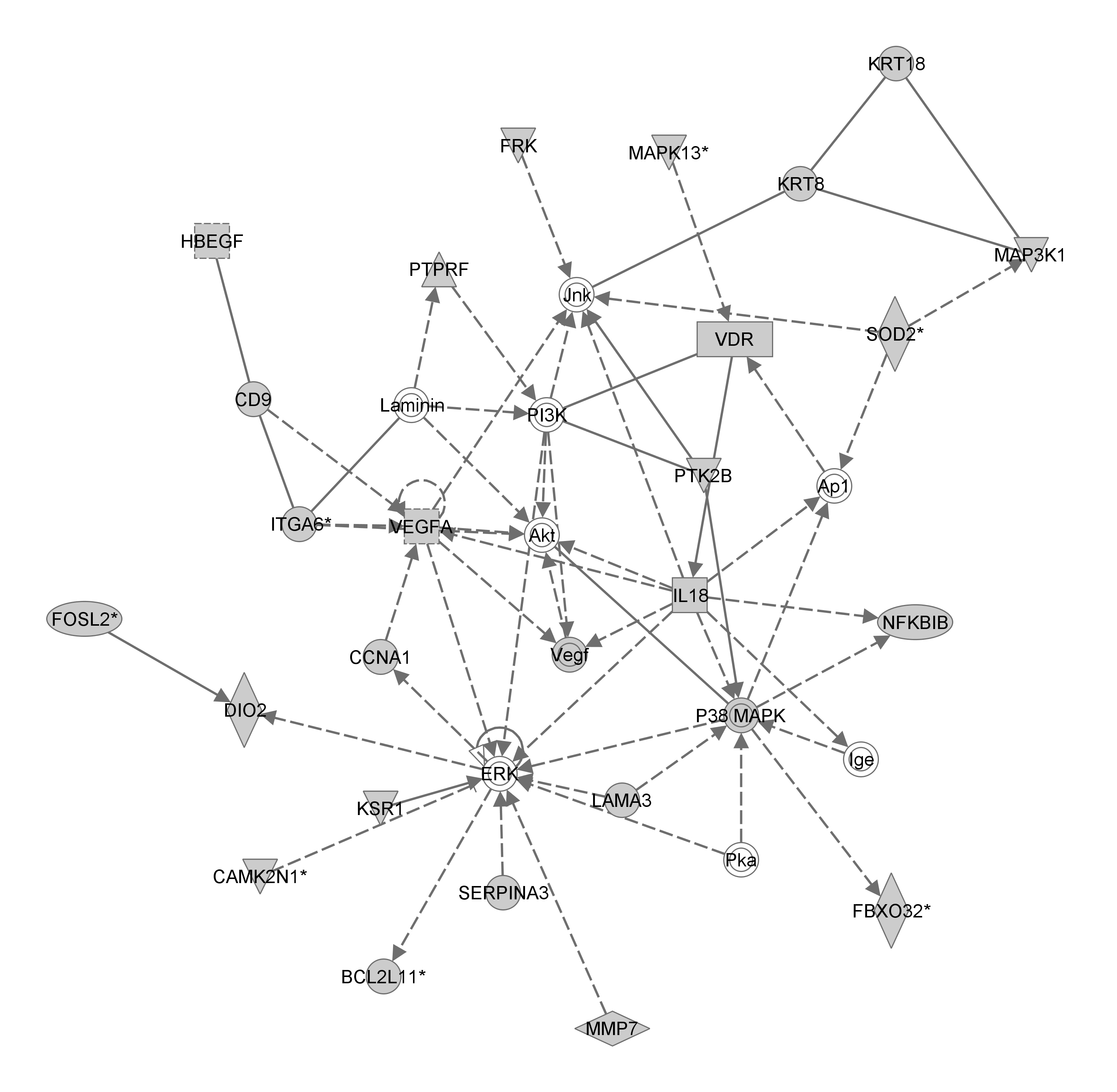

Supplement: Additional file 10 — Figure S8: Ingenuity pathway analysis of genes expressed at lower levels in CD44+/CD24- cells compared to CD44-/CD24+ cells. Genes in this network are linked to PI3 kinase/AKT [file 1471-2407-10-411-S10.JPEG]

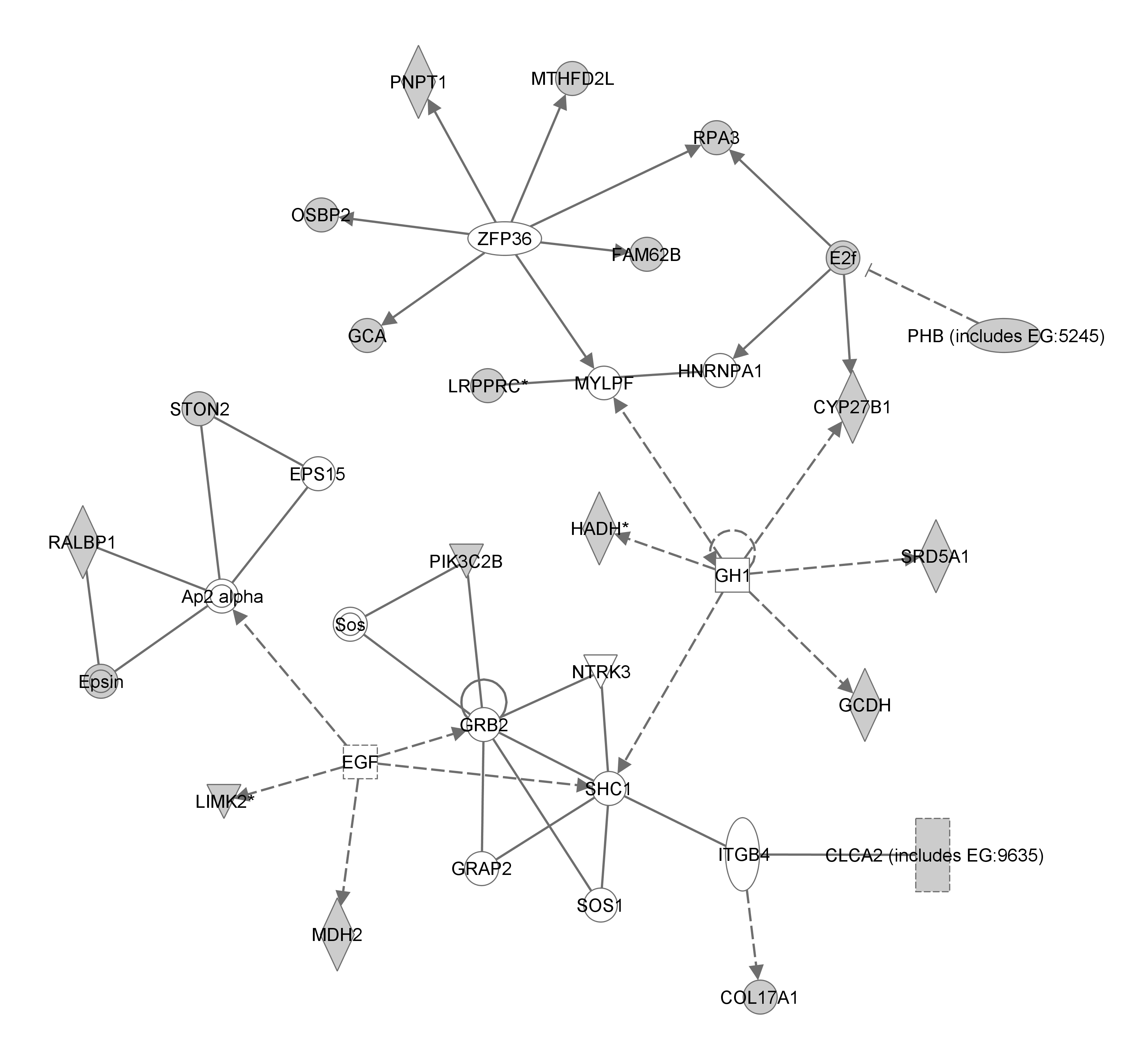

Supplement: Additional file 11 — Figure S9: Ingenuity pathway analysis of genes expressed at lower levels in CD44+/CD24- cells compared to CD44-/CD24+ cells. Genes in this network are linked to multiple signaling molecules including growth hormone (GH1) and GRB2. [file 1471-2407-10-411-S11.JPEG]

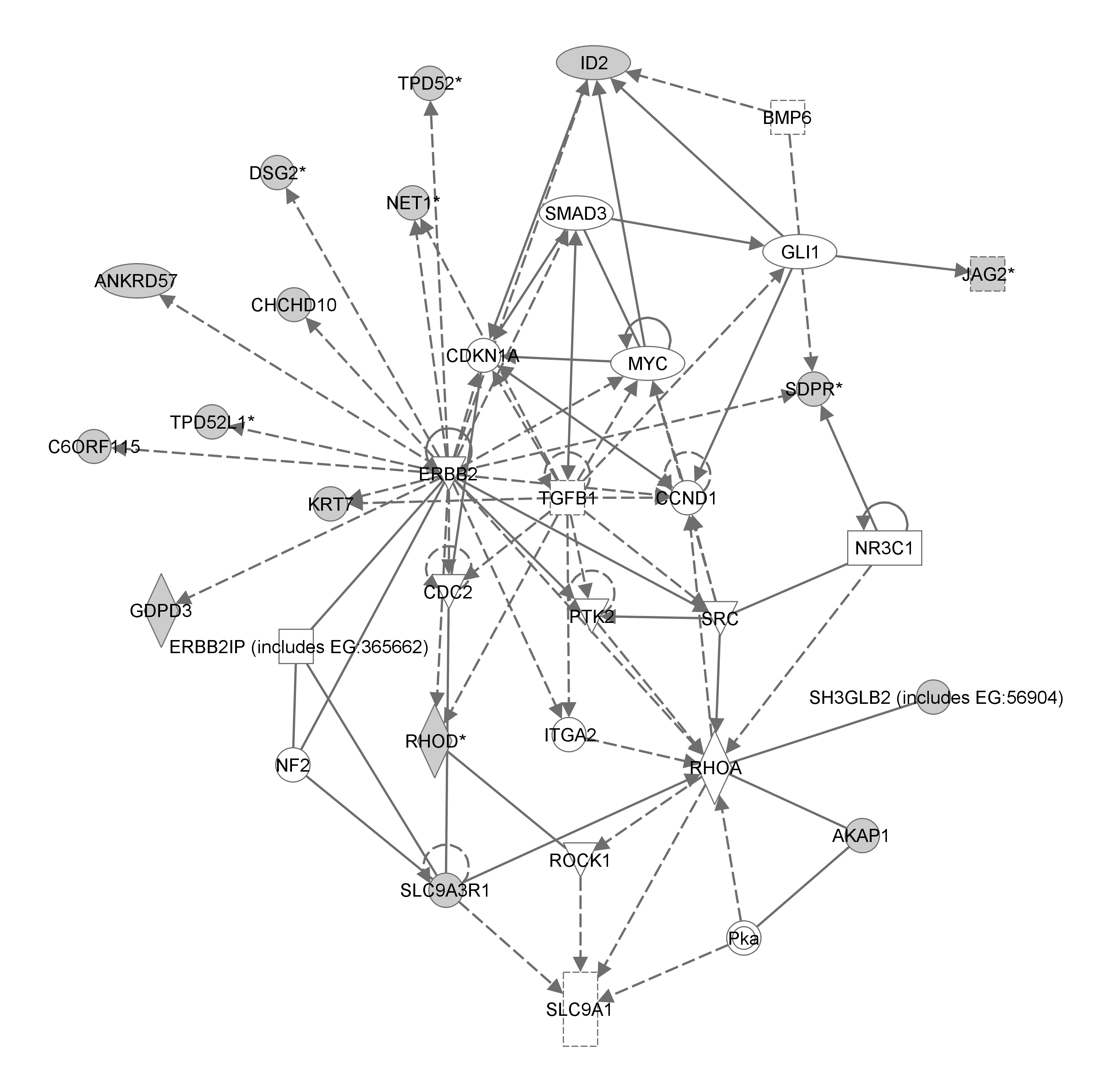

Supplement: Additional file 12 — Figure S10: Ingenuity pathway analysis of genes expressed at lower levels in CD44+/CD24- cells compared to CD44-/CD24+ cells. Genes in this network are linked to ERBB2 and TGFβ1. [file 1471-2407-10-411-S12.JPEG]

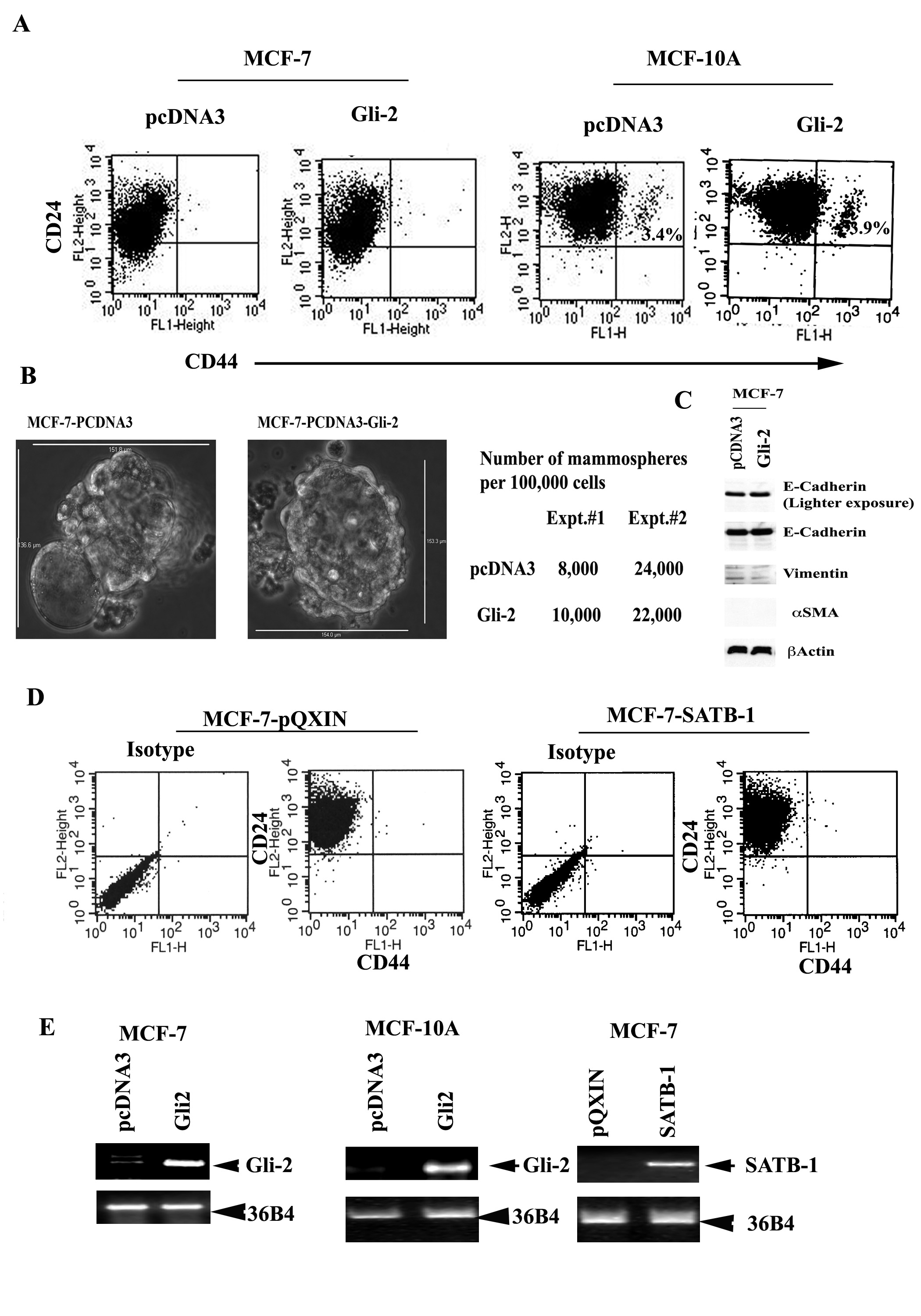

Supplement: Additional file 13 — Figure S11: The effect of Gli-2 and SATB1 overexpression on CD44 and CD24 cell surface expression. This figure shows the inability of Gli-2 and SATB-1 to alter cell surface CD44 and CD24 expression profile, mammosphere formation, and EMT-associated gene expression. [file 1471-2407-10-411-S13.JPEG]
