# Supplementary material for: SLUG/SNAI2 and Tumor Necrosis Factor Generate Breast Cells With CD44+/CD24- Phenotype
Source: BMC Cancer. 2010 Aug 6;10:411. doi: 10.1186/1471-2407-10-411 (PMC3087321; doi:10.1186/1471-2407-10-411)
Supplement: Additional file 14 — Legend for figures and table in additional files. This file provides detailed legend for all figures and tables, particularly Figure S11. [file 1471-2407-10-411-S14.DOC]

**Legend for supplementary data:**

**Figure S1-5:** Ingenuity pathway analyses of genes expressed at higher levels in CD44+/CD24- cells compared to CD44-/CD24+ cells.

**Figure S6-10:** Ingenuity pathway analyses of genes expressed at lower levels in CD44+/CD24- cells compared to CD44-/CD24+ cells.

**Figure S11** Gli-2 and SATB-1 fail to alter CD44 and CD24 cell surface profile. A) CD44 and CD24 cell surface expression profile in pcDNA3 (control vector) and Gli-2 expressing MCF-7 and MCF-10A cells. A modest increase in CD44+/CD24+ cells among MCF-10A-Gli-2 cells is not reproducible. B) MCF-7pcDNA3 and MCF-7-Gli-2 cells give rise to similar number of mammospheres with similar size. C) Gli-2 overexpression did not alter the expression levels of EMT markers in MCF-7 cells. D) SATB-1 fails to alter the CD44 and CD24 profile of MCF-7 cells. E) Gli-2 and SATB-1 levels in overexpressing cells, as assessed by RT-PCR and gel electrophoresis.

Table S1: Sequence of primers used in RT-PCR assays.

Table S2: Differentially expressed genes in MCF-10A CD44+/CD24- and CD44-/CD24+ subpopulation. Numbers with negative values are elevated in CD44+/CD24- cells, whereas numbers with positive values are elevated in CD44-/CD24+ cells.
